# Metastability, fractal scaling, and synergistic information processing: What phase relationships reveal about intrinsic brain activity

**DOI:** 10.1016/j.neuroimage.2022.119433

**Published:** 2022-10-01

**Authors:** Fran Hancock, Joana Cabral, Andrea I. Luppi, Fernando E. Rosas, Pedro A.M. Mediano, Ottavia Dipasquale, Federico E. Turkheimer

**Affiliations:** a Department of Neuroimaging, Institute of Psychiatry, Psychology and Neuroscience, King's College London, London, United Kingdom; b Life and Health Sciences Research Institute (ICVS), School of Medicine, University of Minho, Portugal; c Division of Anaesthesia, School of Clinical Medicine, University of Cambridge; d Department of Clinical Neurosciences, University of Cambridge; e Leverhulme Centre for the Future of Intelligence, University of Cambridge; f Alan Turing Institute, London, United Kingdom; g Centre for Psychedelic Research, Department of Brain Science, Imperial College London, London SW7 2DD, United Kingdom; h Data Science Institute, Imperial College London, London SW7 2AZ, United Kingdom; i Centre for Complexity Science, Imperial College London, London SW7 2AZ, United Kingdom; j Department of Psychology, University of Cambridge, Cambridge CB2 3EB, United Kingdom; k Department of Psychology, Queen Mary University of London, London E1 4NS, United Kingdom

**Keywords:** Functional magnetic resonance imaging, Dynamic functional connectivity, Complexity, Metastability, Fractal scaling, Integrated information, LEiDA, fMRI, functional magnetic Resonance Imaging, BOLD, blood oxygen level dependent, FC, Functional Connectivity, dFC, dynamic Functional Connectivity, LEiDA, Leading Eigenvector Dynamic Analysis, DFA, detrended fluctuation analysis

## Abstract

Dynamic functional connectivity (dFC) in resting-state fMRI holds promise to deliver candidate biomarkers for clinical applications. However, the reliability and interpretability of dFC metrics remain contested. Despite a myriad of methodologies and resulting measures, few studies have combined metrics derived from different conceptualizations of brain functioning within the same analysis - perhaps missing an opportunity for improved interpretability. Using a complexity-science approach, we assessed the reliability and interrelationships of a battery of phase-based dFC metrics including tools originating from dynamical systems, stochastic processes, and information dynamics approaches. Our analysis revealed novel relationships between these metrics, which allowed us to build a predictive model for integrated information using metrics from dynamical systems and information theory. Furthermore, global metastability - a metric reflecting simultaneous tendencies for coupling and decoupling - was found to be the most representative and stable metric in brain parcellations that included cerebellar regions. Additionally, spatiotemporal patterns of phase-locking were found to change in a slow, non-random, continuous manner over time. Taken together, our findings show that the majority of characteristics of resting-state fMRI dynamics reflect an interrelated dynamical and informational complexity profile, which is unique to each acquisition. This finding challenges the interpretation of results from cross-sectional designs for brain neuromarker discovery, suggesting that individual life-trajectories may be more informative than sample means.

## Highlights

• Spatiotemporal patterns of phase-locking tend to be time-invariant.
• Global metastability is representative and stable in a cohort of heathy young adults.
• dFC characteristics are in general unique to any fMRI acquisition.
• Dynamical and informational complexity are interrelated.
• Complexity science contributes to a coherent description of brain dynamics.

## Graphical abstract

**Figure:**
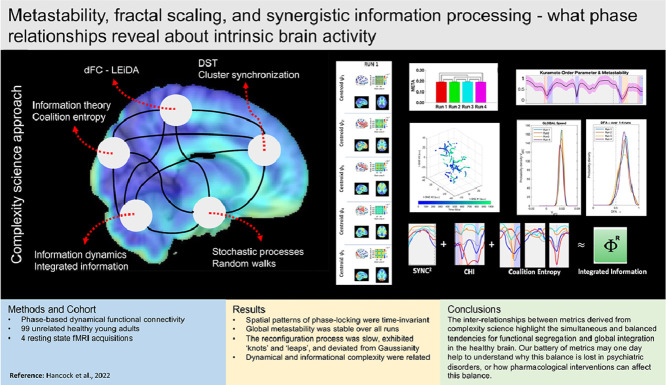

## Introduction

There is great anticipation that functional neuroimaging may complement current clinical phenomenology in the diagnosis of disorders of brain functioning, and provide brain-based markers for patient stratification, disease progression tracking, and prediction of treatment outcomes (Zhang et al., 2021). In this context, the investigation of the brain's functional connectivity (FC) – as revealed by resting-state functional magnetic resonance imaging (fMRI) – holds promise for enabling tools of great clinical value, with thousands of articles per year focused on elucidating properties of normal and abnormal whole-brain functionality (Zhang et al., 2021). Static FC reveals the statistical interdependence among different brain regions using blood oxygenation level dependent (BOLD) signals (Friston, 1994). However, these static measures camouflage the inherent dynamic nature of brain activity, which is captured with time-varying functional connectivity, or dynamic FC (dFC). Unfortunately, the fact that fMRI may be capturing something other than BOLD signals (Drew et al., 2020; Raut et al., 2021), and in the absence of a ground truth, the hurdles to use FC metrics in the clinic are high (Woo and Wager, 2015), and considerably higher for dFC due to issues of interpretation (Lurie et al., 2020) and sampling variability (Laumann et al., 2017), although the latter has been rigorously challenged (Miller et al., 2018). Moreover, the popularity of FC and dFC methods comes with a plethora of heterogeneous methodologies derived from distinct conceptualizations of brain functioning (Bijsterbosch et al., 2020).

Candidate neuromarkers should demonstrate a high degree of reliability and ideally be robust and interpretable in terms of neuroscience (Woo and Wager, 2015). Despite efforts to assess the test-retest reliability of dFC metrics, the results remain contested (Abrol et al., 2017; Bijsterbosch et al., 2017; Choe et al., 2017; Orban et al., 2020; Vaisvilaite et al., 2021; Vohryzek et al., 2020). Common approaches to address these concerns of validity include comparison of results with null models (Battaglia et al., 2020) or replication of results in alternative datasets (Varley et al., 2020). Neuroscientific interpretation of candidate neuromarkers is enhanced with convergence of evidence from multiple sources (Woo and Wager, 2015), and together with reliability, is one of the necessary conditions to introduce neuromarkers into the clinic.

With this in mind, in this paper we took a complexity-science perspective to identify a number of diverse dFC metrics for investigation (Turkheimer et al., 2021). Complexity science takes an inter-disciplinary approach to identify common laws that govern complex systems, bringing together tools from statistical physics, dynamical systems theory, information theory, and other fields (Holland, 2014; Thurner et al., 2018; Waldrop, 1993). Conceptualizing the brain as a complex system offers novel perspectives on spontaneous ongoing brain dynamics (Turkheimer et al., 2021). While the exact definition of a complex system continuously evolves (Ladyman et al., 2013; Turkheimer et al., 2021), the brain satisfies the four shared properties that characterizes a system as ‘complex’ (Jensen, 1998):

1 Multiplicity and interdependence: the brain is made of small subunits that interact with each other through a vast network of local and long-range connections.
2 Nonlinearity: The interactions between neural elements are often nonlinear, giving rise to rich dynamical phenomena.
3 Self-organization: The activity of the multiple brain sub-units develops into structured patterns spontaneously, in the absence of any form of centralized control mechanisms.
4 Emergence: The macroscopic behavior of coordinated brain activity cannot be understood purely in terms of the neuron-to-neuron interactions.

This perspective allows us to investigate the brain with the sophisticated conceptual machinery of complexity science, complementing the existing repertoire of neuroimaging analysis techniques with tools specifically designed to fully exploit the richness of imaging datasets.

The existence of distinct methodologies that investigate intrinsic brain activity either from a dynamical systems perspective, from considerations of the time-evolution of the dynamical system as a stochastic process, or from an information processing perspective, compels us to confront the challenging task of piecing together a coherent description of brain dynamics consistent across the underlying theories.

Two specific metrics, metastability and integrated information, derived from bottom-up and top-down analysis respectively, hold special interest for investigation. Theoretically, metastability has been described as a subtle blend of segregation and integration among brain regions that show tendencies to diverge and function independently, with tendencies to converge and function collectively (Tognoli and Kelso, 2014). Metastability has been considered a key attribute for computational models exploring mechanisms of brain dynamics and an important indicator of healthy brain functioning (Deco et al., 2017). From an alternative but complementary perspective, integrated information (operationalized as the quantity Φ) has been proposed as a way of quantifying the balance between integration and segregation, and possibly consciousness (Tononi, 2004). More recent metrics of integrated information, $\Phi^{R}$, extend this construct to reflect the degree of synergistic and transfer information processing across brain areas (Mediano et al., 2022). Therefore, we sought to investigate if these two metrics contributed converging evidence for the processes of integration and segregation that are believed to take place as part of intrinsic brain activity.

Our objective was to develop a coherent description of brain dynamics consistent across underlying theories. Therefore, rather than investigate metastability and integrated information in isolation, we assessed them in combination with metrics originating in complexity-science, as well as metrics identified theoretically or empirically as characterizing or contributing to metastability or integrated information. Whilst the methodologies used in this study have already been individually validated against null models or with surrogate data (Battaglia et al., 2020; Honari et al., 2021; Mediano et al., 2022), there is a lack of studies where these methodologies were used to compare performance in the same subjects across multiple fMRI acquisitions. Therefore, we set out to answer the following questions: are the chosen dFC metrics representative and reliable across multiple fMRI acquisitions? Are these metrics related via their ability to capture different aspects of dFC? And finally, what are the implications of these relationships?

To address these questions, we used four resting-state fMRI acquisitions recorded on two consecutive days from 99 healthy unrelated participants from the Human Connectome Project (Van Essen et al., 2013), and considered an anatomical parcellation with and without the cerebellar regions.

## Materials and methods

### Data

All data used in this study was collected for the Human Connectome Project, WU-Minn Consortium (Principal Investigators: David Van Essene and Kamil Ugurbil; 1U54MH091657) with funding from the sixteen NIH Institutes and Centers supporting the NIH Blueprint for Neuroscience Research; and by the McDonell Center for Systems Neuroscience at Washington University.

### Ethics statement

The Washington University institutional review board approved the scanning protocol, participant recruitment procedures, and informed written consent forms, and consented to share deidentified data.

### Participants

We used the data from the ‘500 subject’ release but restricted our analysis to the ‘100 Unrelated Subjects’ (aged 20 to 35 years old, 54 females (Glasser et al., 2013)). A list of employed subject ID numbers and associated scan times is provided in Supplementary Table ST1.

### fMRI data acquisition and pre-processing

Each participant underwent four scans of resting-state fMRI (rs-fMRI) collected over two experimental sessions (two scans in each session) which took place on consecutive days. The datasets acquired from all participants in each of the 4 scans are referred to as ‘runs’ 1 to 4. During each scan 1200 frames were acquired using a multiband sequence at 2 mm isotropic resolution with a repetition time (TR) of 0.72 s over the span of 14 min 24 s. Participants were instructed to maintain fixation on a bright crosshair presented on a dark background in a darkened scanning room. The two scans in each session differed only in the oblique axial acquisition phase encoding. For the first 6 subjects, the rs-fMRI runs were acquired using a Right-Left (RL) phase-encoding followed by a Left-Right (LR) phase-encoding on both days. For the following 94 subjects, the order of the different phase-encoding acquisitions for the rs-fMRI runs across days was counterbalanced (RL followed by LR on Day 1; LR followed by RL on Day 2).

Data were pre-processed with the HCP's minimal pre-processing pipeline, and denoising was performed by the ICA-FIX procedure (Glasser et al., 2013; Griffanti et al., 2014; Salimi-Khorshidi et al., 2014). A complete description of the acquisition and pre-processing details may be found at the HCP website https://www.humanconnectome.org/. Briefly, fMRI data was gradient-nonlinearity distortion corrected, rigidly realigned to adjust for motion, fieldmap corrected, aligned to the structural images, and then registered to MNI space with the nonlinear warping calculated from the structural images. ICA-FIX was then applied on the data to identify and remove motion and other artifacts in the timeseries. The resulting files provided the baseline for this study (e.g., MNINonLinear/Results/rfMRI_REST1_RL/rfMRI_REST1_RL_hp2000_clean.nii.gz from released HCP data).

One subject was excluded from the analysis as the image file was corrupted.

### Parcellations

We parcellated the pre-processed fMRI data by averaging time-courses across all voxels for each region defined in the anatomical parcellation AAL (Tzourio-Mazoyer et al., 2002) considering all cortical and subcortical brain areas including the cerebellum, *N* = 116 or without the cerebellum *N* = 90. We choose the AAL parcellation as it includes subcortical and cerebellar regions which are relevant for application of the methods in future studies with psychiatric cohorts (Anteraper et al., 2021; Cierpka et al., 2017; Duan et al., 2015; McCutcheon et al., 2019, McCutcheon et al., 2020).

### Bandpass filtering

To isolate low-frequency resting-state signal fluctuations, we bandpass filtered the parcellated fMRI time-series within 0.01–0.08 Hz using a discrete Fourier transform (DST) computed with a fast Fourier transform (FFT) algorithm in MATLAB. We included frequencies below 0.03 Hz, as unpublished data indicate that results obtained in the lower frequency bands are consistent with those found in the 0.01–0.08 Hz band (see inline Supplementary Figure S1). Additionally, we applied Carson's empirical rule (Carson, 1922; Pachaud et al., 2013) on the analytical signal which was calculated using the Hilbert transform of the real signal (Gabor, 1946), to confirm non-violation of the Bedrosian theorem for our bandpassed signals (see inline Supplementary Figure S2).

### Phase synchronization measures

We investigated two complementary phase synchronization measures based on phase and phase difference. Instantaneous phase θ(t) is obtained from the analytical signal. We define phase synchrony (PS) as the magnitude of the Kuramoto order parameter (Acebrón et al., 2005; Cabral et al., 2011; Deco et al., 2017, Deco et al., 2018; Shanahan, 2010). This has also been referred to by other authors as Phase Coherence (Breakspear et al., 2010; Váša et al., 2015). For any brain region *M of r parcels* at time *t*, PS is then defined as

(1) $$PS_{M}\left(t\right)={\left|\langle e^{i\theta \left(r,t\right)}\rangle \right|}_{,\;r\in M}$$

Phase synchrony measures the degree of synchronization across the brain region *M,* at time *t* and is bound between 0 and 1.

Phase synchrony is distinct from phase coherence (PC) as used in (Pedersen et al., 2018). PC is related to phase difference, is measured as (1−|sin(Δθ(t)|), and is bound between 0 and 1. PC was not used in this study.

We define phase-locking (PL) as the cosine of the relative phase (Alonso Martínez et al., 2020; Cabral et al., 2017; Deco et al., 2019, 2017a; Figueroa et al., 2019; Honari et al., 2021; Lord et al., 2019; Vohryzek et al., 2020)

PL(n,p,t)=cos(Δθ(n,p,t))

where

(2) $$\begin{array}{ccc} & & \Delta \theta \left(t\right)=\left(\theta \left(n,t\right)-\theta \left(p,t\right)\right),\;\text{the}\;\text{phase}\;\text{difference}\;\text{between}\;\text{regions}\;\text{n}\;\text{and}\;\text{p}. \end{array}$$

Phase-locking is a measure of synchronization that preserves positive and negative dependence in the data, that is, in-phase locking and anti-phase locking. Phase locking is bound between −1 and +1.

Phase-locking is distinct from phase locking value (PLV) as used in (Ponce-Alvarez et al., 2015). PLV measures the magnitude (absolute value) of the Δθ(t) and is bound between 0 and 1, and was not used in this study.

**Fig. 1:**
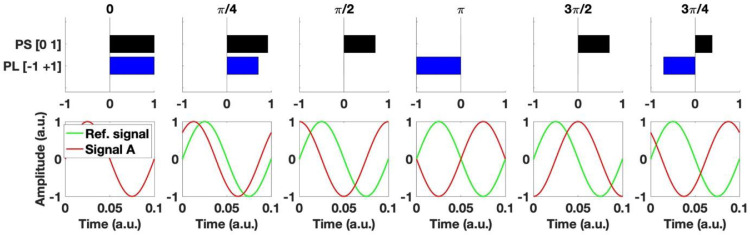
Two complementary phase synchronization measures for the calculation of dFC metrics. Phase synchrony (PS) reflects the degree of synchronization between the two signals, whilst phase-locking (PL), evaluated as the cosine of the relative phase, reflects phase alignment and is sensitive to both in-phase and anti-phase relationships between the two signals.

The behavior of phase synchrony and phase-locking between 2 signals is illustrated in Fig. 1, where the phase of one signal (A) shifts away from the phase of a reference signal.

### Functional connectivity through phase-locking

We estimated functional connectivity (FC) with the nonlinear measure of phase-locking which may be more suitable than linear measures such as Pearson correlation for analyzing complex brain dynamics. Specifically, phase synchronization measures are not affected by amplitude variability between subjects (Glerean et al., 2012), and nonlinear methods provide insight into interdependence between brain regions at both short and large time and spatial scales allowing the analysis of complex nonlinear interactions across space and time (Pereda et al., 2005; Quian Quiroga et al., 2002). From a practical perspective, unlike correlation or covariance measures, phase synchronization can be estimated at the instantaneous level and does not require time-windowing. When averaged over a sufficiently long time window, phase-locking values provide a close approximation to Pearson correlation, varying within the same range of values (Cabral et al., 2017; Honari et al., 2021).

Indeed, a variety of phase synchronization measures have been leveraged in dFC studies to date. An overview presenting the range of studies, and the phase synchronization measure assessed in those studies, is presented in Supplementary Table ST2.

Following (Cabral et al., 2017), we first calculated the analytical signal using the Hilbert transform of the real signal (Gabor, 1946). Then, the instantaneous phase-locking between each pair of brain regions *n* and *p* was estimated for each time-point *t* as the cosine difference of the relative phase as

(3) iPL(n,p,t)=cos(θ(n,t)−θ(p,t))

Phase-locking at a given timepoint ranges between −1 (regions in anti-phase) and 1 (regions in-phase). For each subject the resulting *iPL* was a three-dimensional tensor of size *NxNxT* where *N* is the dimension of the parcellation, and *T* is the number of timepoints in the scan.

### LEiDA – leading eigenvector dynamic analysis

To reduce the dimensionality of the phase-locking space for our dynamic analysis, we employed the Leading Eigenvector Dynamic Analysis (LEiDA) (Cabral et al., 2017) method. The leading eigenvector *V_1_(t)* of each *iPL(t)* is the eigenvector with the largest magnitude eigenvalue and reflects the dominant FC (through phase-locking) pattern at time *t. V_1_(t)* is a *Nx1* vector that captures the main orientation of the fMRI signal phases over all anatomical areas. Each element in *V_1_(t)* represents the projection of the fMRI phase in each region into the leading eigenvector. When all elements of *V_1_(t)* have the same sign, this means that all fMRI phases are orientated in the same direction as *V_1_(t)* indicating a global mode governing all fMRI signals. When the elements of *V_1_(t)* have both positive and negative signs, this means that the fMRI signals have different orientations, behaving like opposite anti-nodes in a standing wave. This allows us to separate the brain regions into two ‘communities’ (or poles) according to their orientation or sign, where the magnitude of each element in *V_1_(t)* indicates the strength of belonging to that community (Newman, 2006). For more details and graphical representation see (Figueroa et al., 2019; Lord et al., 2019; Vohryzek et al., 2020). The outer product of *V_1_(t)* reveals the FC matrix associated with the leading eigenvector at time t.

### Mode extraction

To identify recurring spatiotemporal modes ψ or phase-locking patterns, we clustered the leading eigenvectors for each run with K-means clustering with 300 replications and up to 400 iterations for 2–10 centroids considering 116 and 90 (i.e., excluding the cerebellum) anatomical regions. K-means clustering returns a set of K central vectors or centroids in the form of *Nx1* vectors *V_c_*. As *V_c_* is a mean derived variable, it may not occur in any individual subject data set. To obtain time courses related to the extracted modes $\psi_{k}$ at each TR we assign the cluster number to which *V(t)* is most similar using the cosine distance.

### Mode visualization

We rendered the centroid vectors *V_c_* in cortical space by representing each element as a sphere placed at the center of gravity of the relevant brain region, and scaling the color of the spheres according to the value of the relevant eigenvector. Regions with similar phase orientation are colored alike (yellow-to-red for the smallest community and cyan-to-blue for the largest community), where darker colors (red/blue) indicate weak contributions and lighter colors (cyan/yellow) indicate stronger contributions. We also plot links between the corresponding areas to highlight the network formed by the smallest community of brain areas (see Supplementary Figure S5).

### Cluster representation in voxel space

To obtain a visualization in voxel space of the spatial modes *V_c_* we first reduced the spatial resolution of all fMRI volumes from 2mm^3^ to 10mm^3^ to obtain a reduced number of brain voxels (here *N* = 1821*)* to be able to compute the eigenvectors of the *NxN* phase-locking matrices. The analytic signal of each 10mm^3^ voxel was computed using the Hilbert transform, and the leading eigenvectors were obtained at each time point (with size *NxT*). Subsequently, the eigenvectors were averaged across all time instances assigned to a particular cluster, obtaining in this way, for each cluster, a *1xN* vector representative of the mean phase-locking pattern captured in voxel space.

### Measures and metrics

The following sections provide an accessible overview of the measures and metrics used in this study. Detailed mathematical treatment and explanations for all metrics may be found in Supplementary methods and metrics. Each metric has found application in either theoretical or empirical studies, or both. Examples of their application may be found in Supplementary Table ST3.

### Metrics derived from phase-locking

Fractional occurrence of mode $\psi_{k}$ was calculated as number of timepoints assigned to mode $\psi_{k}$ divided by the total number of timepoints. This measure reflects the proportion of time the fMRI activity patterns are closer to mode $\psi_{k}$ than to any other mode $\psi_{\neq k}$. Its values are bound between 0 and 1.

Duration of mode $\psi_{k}$ was calculated as the mean of all consecutive periods spent in a particular mode, measured in seconds.

Reconfiguration speeds were calculated as 1 – correlation between functional connectivity (*iPL* matrices) at time *t* and *t* *+1.* This measure characterizes the time evolution of the phase-locking modes. Low speed indicates smooth transitions in phase-locking relationships. Faster speed indicates abrupt switching between phase-locking relationships.

The fractal scaling coefficient α derived from detrended fluctuation analysis (DFA) characterizes power-law scaling in a time series. DFAα values less than 0.5 indicate non-persistent fluctuations and a return to the mean. Values equal to 0.5 indicate random fluctuations and an underlying process with no memory. Values between 0.5 and 1 indicate persistent fluctuations and an underlying process that has memory and long-term correlations.

Following Ton and Daffertshofer (2016), power-law scaling was tested for linearity using a Bayesian model comparison technique and the best fit model was selected with Bayesian Information Criterion. Only subjects that exhibited extended linear power-law scaling were included in the summary metric of DFAα.

### Metrics derived from phase synchrony

Empirical metastability studies to date have used pre-defined resting-state networks (RSN) extracted with ICA (Hellyer et al., 2014), with network masks (Lee et al., 2018), or with functional templates (Lee et al., 2019) to represent communities of oscillators for investigation of network synchrony and metastability. In contrast, we decided to take a purely data driven approach, using the recurrent modes extracted with K-means clustering to represent communities of oscillators. As we decided to retain 5 recurrent modes (see Results), we therefore have 5 communities of oscillators $\psi_{1}$ - $\psi_{5}$. Note that the AAL regions are not constrained to a single community and so the communities reflect time-varying coalitions among regions.

Synchronization was calculated as the time-average of the Kuramoto order parameter in each community, which is given by

(4) $$Z_{\psi }\left(t\right)=\langle e^{i\theta \left(r,t\right)}\rangle ,{\;}_{r\in \psi_{\;}\;}$$

Above, $Z_{\psi }\left(t\right)$ is a complex value where its magnitude, $\;SYNC_{\psi }$= $|Z_{\psi }\left(t\right)|,$ provides a quantification of the degree of synchronization of the community at each time *t,* taking values between 1 (for fully synchronized systems) and 0 (for fully desynchronized systems)*.*

Metastability was calculated as the standard deviation over time of the Kuramoto order parameter in each community. The mean value of this measure across communities denoted as global metastability, represents the overall variability in the synchronization across communities.

Cluster synchronization, or chimerality, was calculated as the variance over communities of the Kuramoto order parameter at time *t*. This metric reveals if some communities cluster together in synchrony whilst other communities remain disordered.

Instantaneous phase coherence across communities was calculated as the average phase across communities with synchronization values higher than a synchronization threshold λ > 0.8 at time *t*. This measure represents the coherence between communities when they are highly synchronized internally. Phase coherence coefficient was calculated as the fraction of time that instantaneous phase coherence occurred.

Although chimerality reflects the prevalence of cluster synchronization, it does not indicate if the system repeatedly visits a small number of chimera configurations, or if the system has a large repertoire of such configurations. To quantify the diversity of cluster synchronization, we calculate the coalition entropy formed at each timepoint *t,* reported in bits (Mediano et al., 2022, Mediano et al., 2016; Shanahan, 2010; Wildie and Shanahan, 2012).

Integrated information $\Phi^{R}$ (Mediano et al., 2021) was computed from 5 binarized time-series, one for each mode extracted through K-means clustering. Values were set to 1 if synchronization values were higher than a coalition threshold λ > 0.8 at time *t*. The value for λ was chosen based on results from computational studies (Mediano et al., 2022, Mediano et al., 2016) and is identical to the synchronization threshold explained earlier. In this study, integrated information indicates the degree of synergistic and transfer information processing within the system computed over an integration timescale τ reported in bits.

### Statistical analysis

#### Interclass correlation coefficient (ICC)

ICC is a relative metric that is used for test-retest reliability in measurement theory (Lavrakas, 2008). It is generally defined as the proportion of the total measured variance that can be attributed to within subject variation. As such, ICC coefficients may be low when there is little variance between subjects, that is in a homogeneous sample, or when the within-subject variance is large (Xing and Zuo, 2018). In this study we use the ICC forms from Shrout and Fleiss (1979).

There are many scales for ICC, so for clarity we will use those of (Landis and Koch, 1977):

• low (0 < ICC < 0.2)
• fair (0.2 < ICC < 0.4)
• moderate (0.4 < ICC < 0.6
• substantial (0.6 < ICC < 0.8)
• almost perfect (0.8 < ICC < 1)

We calculated the run reliability of mode ψ extraction with ICC(1,1) in search of agreement rather than consistency across runs (Noble et al., 2021). For the test-retest assessment of metric consistency over runs, we used the ICC(3,1) form (Shrout and Fleiss, 1979) as recommended by (Koo and Li, 2016), which is the equivalent of a 2-way mixed ANOVA (Li et al., 2015). As such, there is an assumption that the data comes from a normal distribution. When the assumption of normality is violated, it is recommended to use non-parametric tests such as permutation testing.

#### Repeated measures ANOVA

We performed repeated measures ANOVA on global metrics using the *ranova()* function in MATLAB MathWorks R2021b. Greenhouse-Geisser correction was necessary as the assumption of sphericity was violated in most cases. We therefore assessed normality of the data with Shapiro-Wilk tests and include the results in inline Supplementary Table ST4.

As the results indicated non-normal distribution of the data, we decided to replace ICC(3,1) with non-parametric permutation testing. We also performed repeated measures ANOVA on the mode-specific metrics. It should be noted that for the AAL parcellation that included the cerebellar regions, the order of the modes in run 2 and 4 was adjusted to match the order in run 1 and 3 for all statistical testing.

#### Permutation testing

We used a non-parametric permutation-based paired *t*-test to identify significant differences between runs. This non-parametric two-sample hypothesis test uses permutations of group (run) labels to estimate the null distribution rather than relying on the *t*-test standard distributions. The null distribution was computed independently for each run. A *t*-test was then applied with 1000 permutations to compare runs.

#### Linear mixed effects modeling

We used lmerTest (Kuznetsova et al., 2017) in RStudio 2021.09.1 Build 372, with the purpose of building predictive models with both standardized and non-standardized metric data that could deal with data that was not independent and identically distributed. To investigate the relationship between integrated information and all other metrics, we fitted a linear mixed-effect model (estimated using REML and nloptwrap optimizer) to predict Φ^*R*^ with standardized metric values. The model included RUN as random effect (formula: ∼1 | RUN). 95% Confidence Intervals (CIs) and p-values were computed using the Satterthwaite's method.

### Code availability statement

The Matlab and R code developed for this analysis is available at github.com/franhancock/Complexity-science-in-dFC together with the 5 phase-locking mode centroids for AAL parcellation in NIFTI and in Matlab format.

## Results

### Reliability of dFC measures and metrics

#### Spatial patterns of phase-locking are invariant across fMRI acquisitions

**Fig. 2:**
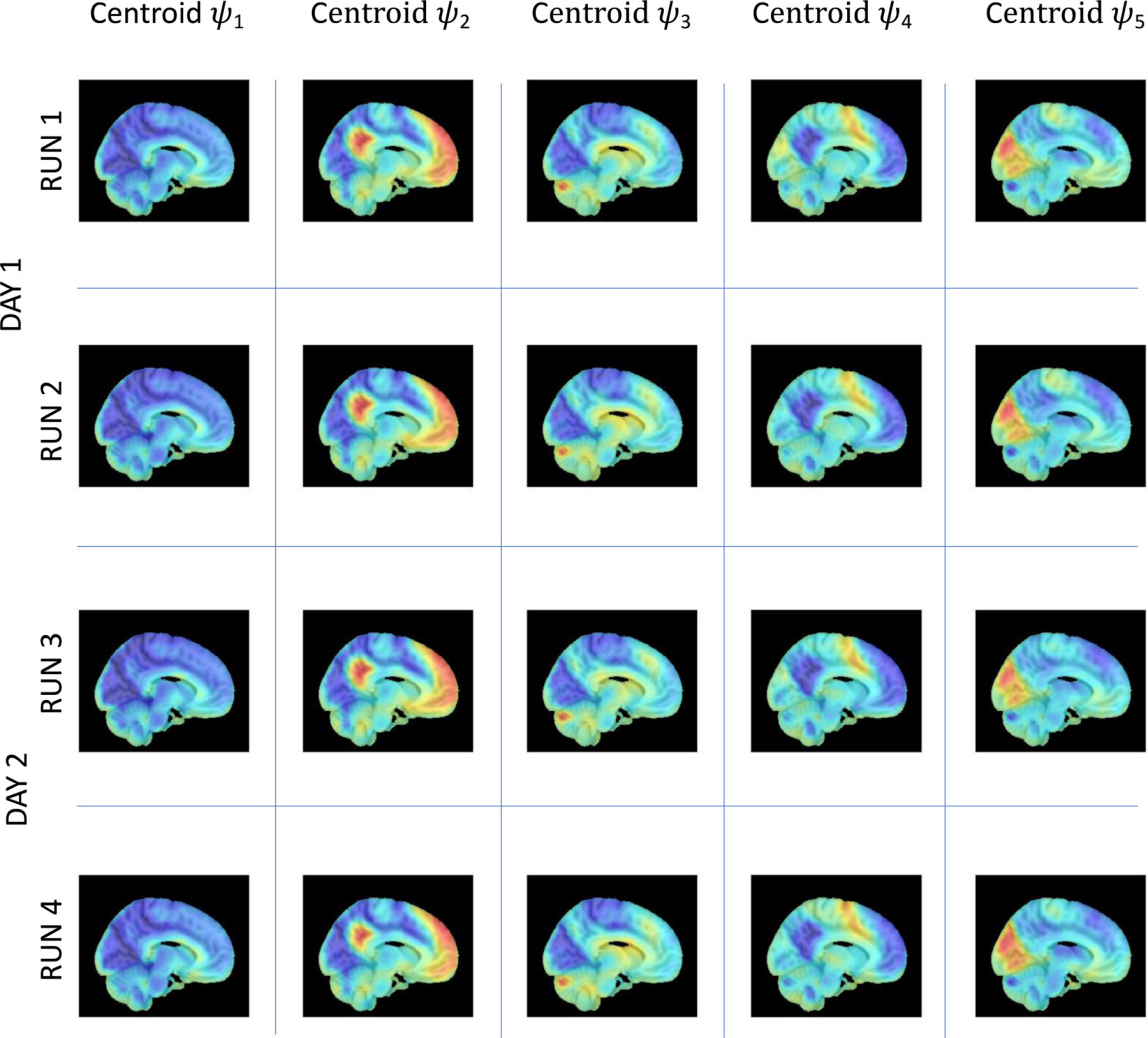
Invariant spatiotemporal patterns of phase-locking obtained independently in each of the 4 fMRI runs on the same 99 participants – sagittal view. LEiDA was applied separately to the 4 fMRI runs recorded on 2 consecutive days from 99 participants and the centroids obtained from clustering into *K* = 5 are reported here. Each centroid V_c_ (with size 1xN, with *N* = K116) is represented for each mode in 10 mm voxel space by averaging the eigenvector values over all time instances assigned to a particular cluster/mode. The modes were then plotted over a 1mm^3^ MNI T1 image.

We first sought to evaluate if the spatiotemporal patterns of phase-locking observed in fMRI are representative and stable across multiple acquisitions. For this purpose, we compared the spatial patterns of phase-locking extracted independently for each of the 4 fMRI runs recorded from the same 99 participants (Fig. 2). Each mode of phase locking $\psi_{K}\;$corresponds to a 1xN vector (with N being the number of brain areas considered) obtained through K-means clustering of phase-locking patterns obtained at every time point in each run. We chose *K* = 5 modes according to the silhouette value (Supplementary Figure S3) and in consideration of a previous test-retest study performed on the same dataset (Vohryzek et al., 2020), and a reproducibility test study on a more extensive dataset with 28 groups of 250 age matched subjects (Abrol et al., 2017). We calculated run reliability with inter-class correlation coefficient (ICC(1,1)) in search of agreement across runs (Noble et al., 2021). With *N* = K90 anatomical non-cerebellar brain regions defined in the AAL parcellation, the modes extracted independently in each run showed almost perfect agreement between runs with 0.99 > ICC > 0.97. With the inclusion of cerebellar regions, the reliability of spatial patterns showed again almost perfect agreement between runs with 1 > ICC > 0.94, although the probability of occurrence differed across runs, altering the order of the modes when sorted by relative occupancy.

**Fig. 3:**
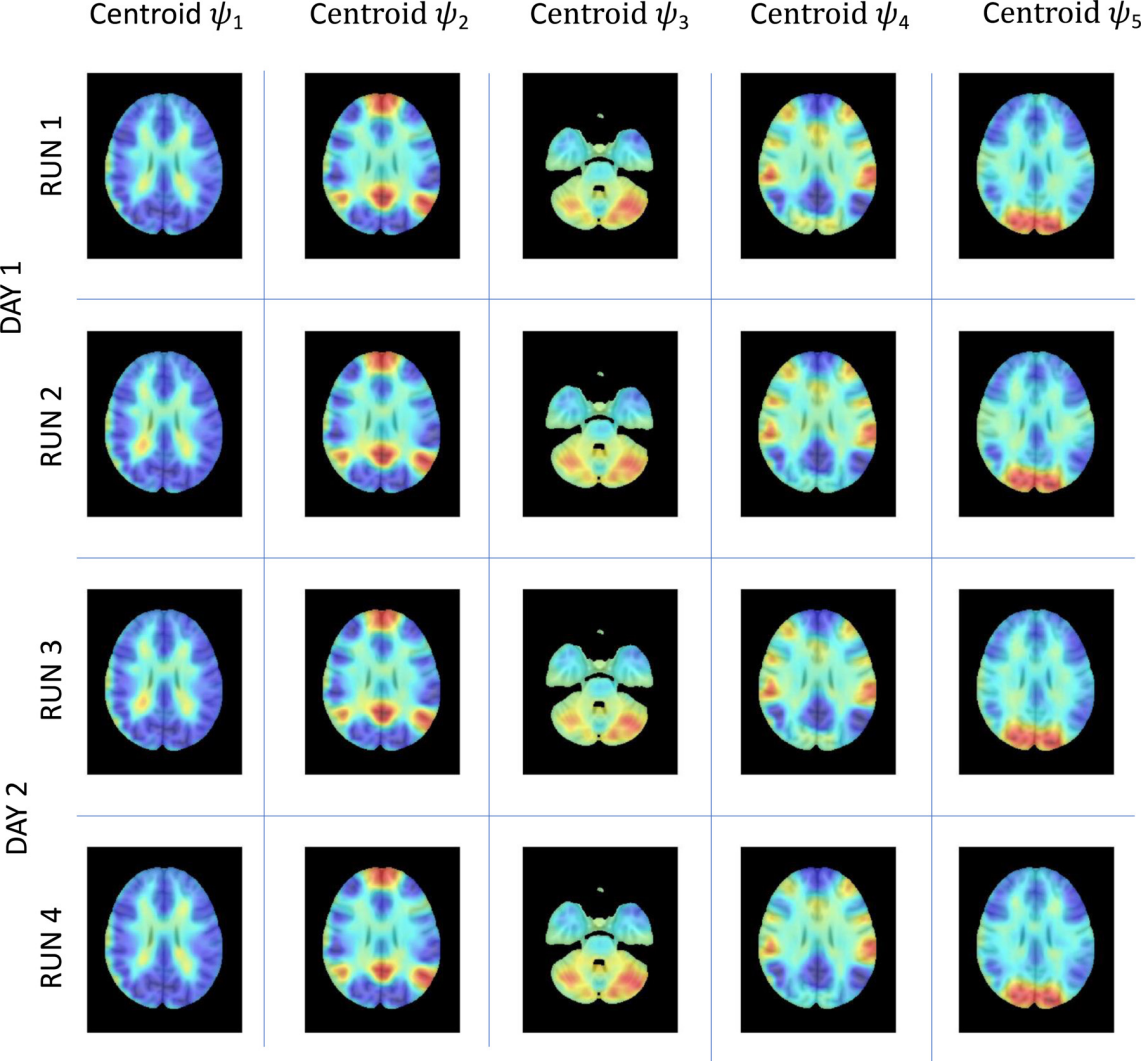
Invariant spatiotemporal patterns of phase-locking obtained independently in each of the 4 fMRI runs on the same 99 participants – axial view. LEiDA was applied separately to the 4 fMRI runs recorded on 2 consecutive days from 99 participants and the centroids obtained from clustering into *K* = 5 are reported here. Each centroid V_c_ (with size 1xN, with *N* = 116) is represented for each mode in 10 mm voxel space by averaging the eigenvector values over all time instances assigned to a particular cluster/mode. The modes were then plotted over a 1mm^3^ MNI T1 image.

The similarity of the 5 cluster centroids $\psi_{k=1,..,5}$ across the 4 runs is clearly visible in Fig. 2 and 3. To illustrate the patterns of phase relationships between brain regions, the 1xN centroids are rendered in cortical space together with the associated phase-locking matrices. In addition, to visualize the phase-relationships in voxel space, we reduce the fMRI volumes from 2mm^3^ to 10mm^3^, resulting in 1821 brain voxels within the MNI brain mask, and compute the eigenvectors of phase-locking at each time point. Subsequently, the eigenvectors are averaged across all time points assigned to each cluster, and represented in sagittal and axial planes overlaying on a 1mm^3^ MNI structural image. This approach allows visualizing the patterns of phase relationships in voxel space, revealing meaningful functional subsystems overlapping with resting-state networks described in the literature. ICC values for both 90 and 116 regions are reported in inline Supplementary Figure S4.

We used the eigenvectors obtained with 116 regions to shed light on the composition of the extracted modes and their putative membership of seven cerebral intrinsic functional networks (Yeo et al., 2011) collectively known as resting-state networks (RSN), and connections with the sub-cortical and cerebellum regions. In inline Supplementary Figure S5 we show the composition of each mode eigenvector color-coded according to the RSNs, and the rendering of these eigenvectors in cortical space.

We find that mode $\psi_{1}\;$represents a global mode where the fMRI signals in all regions are aligned in-phase. Mode $\psi_{2}$ consists of a phase-locking pattern where regions associated with the Default Mode Network (DMN), the Limbic network (LBC), the subcortical hippocampi (SC) regions, and some cerebellum (CB) regions are shifted in phase with respect to the rest of the brain. Mode $\psi_{3}$ comprises regions associated with the Frontal Parietal Area (FPA), the LBC, the SC Caudate and Putamen, and a number of CB regions. Mode $\psi_{4}$ comprises of regions associated with the Sensory Motor network (SMT), and the Ventral Attention network (VAT), with some contribution from the FPA and the CB regions. Finally, $\psi_{5}$ is comprised mainly of the Visual network (VIS) with significantly lower contributions from SMT, LBC, DMN, and SC.

Overall, these results show that spatiotemporal patterns of phase-locking are representative and stable across multiple fMRI acquisitions. They therefore provide a stable basis for the characterization and analysis of our battery of dFC metrics.

#### Global metastability was the most stable metric across all runs

**Fig. 4:**
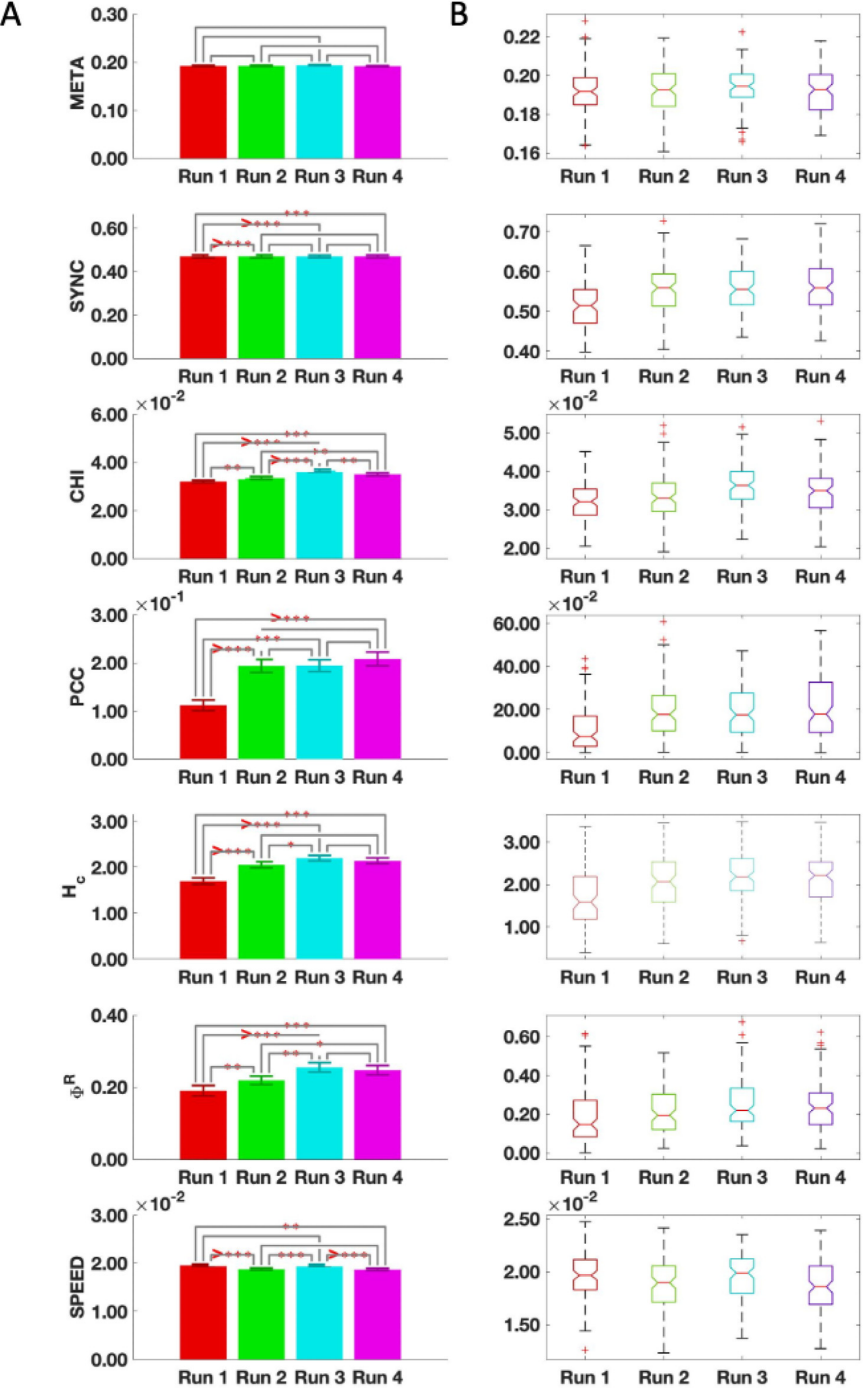
Stability of global metrics across 4 runs. (A) The mean values for each metric are shown as bar plots. The *** indicate a statistically significant difference between the metric across the associated runs where _*_p < 0.05, _**_p < 0.01, _***_p < 0.001, and >_***_p < 0.0001. (B) The distribution of the global metrics across runs. META, metastability; SYNC, synchronization; CHI, chimera index; PCC, phase-coherence coefficient; H_C_ coalition entropy; $\Phi^{R}$, integrated information; SPEED, typical reconfiguration speed.

As a second step, we sought to investigate the stability of a series of global metrics - namely metastability, synchronization, chimera index, phase-coherence coefficient, coalition entropy, integrated information, and typical reconfiguration speed – across different multiple fMRI acquisitions. For this, the values of each metric in four different runs were compared using a non-parametric permutation-based paired *t*-test to identify significant differences. Fig. 4A shows the bar plots for each metric including the mean value and indicators for where significant differences were found between the runs. In Fig. 4B we show the distribution of the metrics across runs which provides complementary information on the median and spread of the metric values across runs.

There was no statistically significant difference in the measure of global metastability across the 4 fMRI runs. When the cerebellum was excluded, however, global metastability did not show the same reliability (inline Supplementary Figure S6). The measures of global synchronization and phase-coherence coefficient were found to be reliable across runs 2, 3, and 4. The remaining metrics however, showed statistically significant differences across the 4 acquisitions.

#### High dynamical and informational complexity across acquisitions of resting-state fMRI

**Fig. 5:**
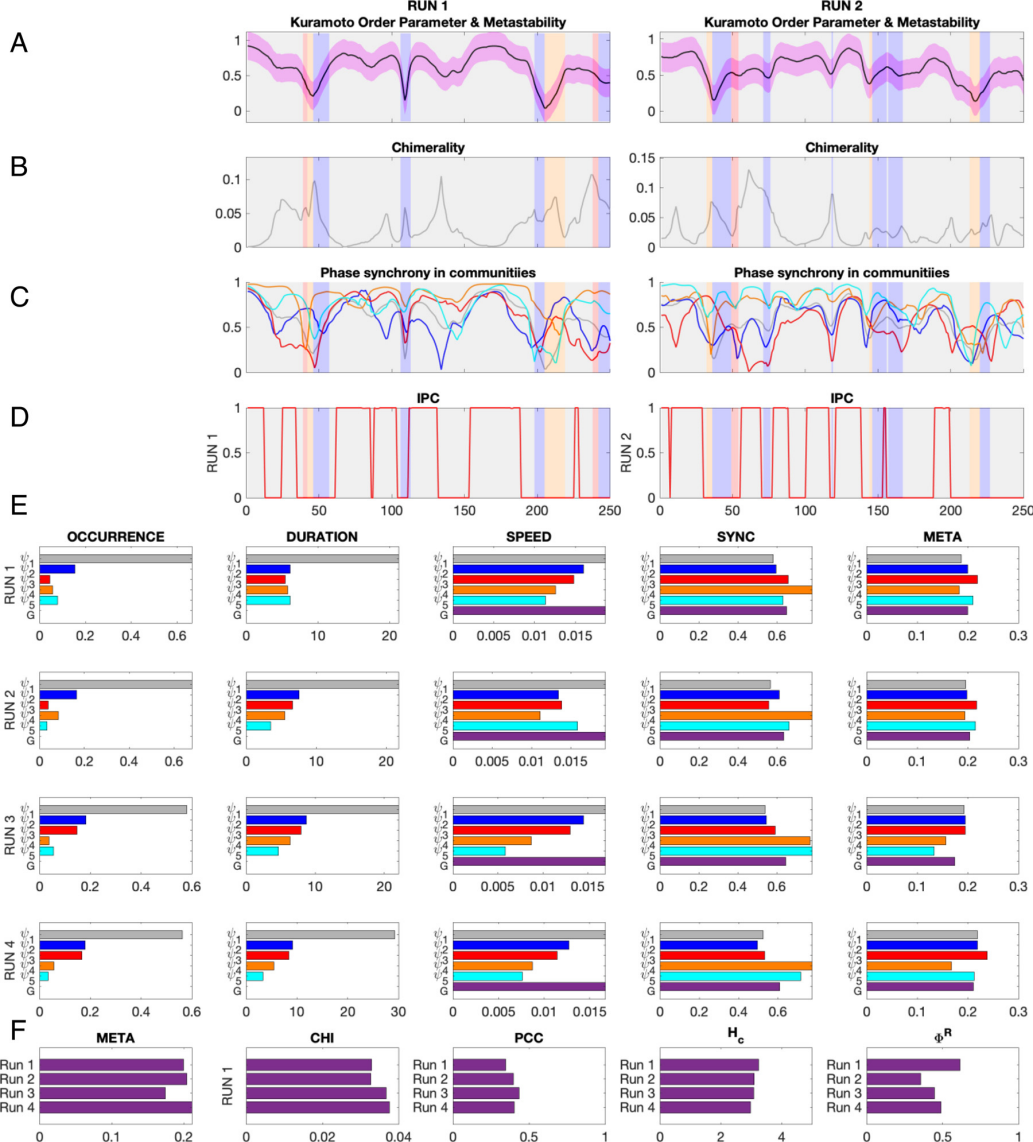
Overview of all metrics in all runs for a representative subject. (A) Exemplar snippets from the instantaneous phase synchrony or Kuramoto order parameter time series for each run color-coded to show which mode was dominant over time. (B) The same as A but for chimerality or cluster synchronization. (C) The evolution of instantaneous synchrony within each of the color-coded modes. (D) The evolution of instantaneous phase-coherence. (E) Mode-specific metrics calculated independently for each of the 4 runs. (F) The values of the global metrics across all 4 runs. META, metastability; CHI, chimera index; PCC, phase coherence coefficient; H_c,_coalition entropy; and $\Phi^{R}$, integrated information.

Although a measure of global metastability was found to be stable across the cohort of healthy young adults between all runs, this was not the case for individual subjects. To illustrate this, we plot the temporal evolution of a series of metrics for two scans and chart the global metrics for each of the 4 scans from one representative subject as illustrated in Fig. 5. As is evident in Fig. 5F, the measure for global metastability, META, is lower in RUN 3 than any of the other runs. For comparison purposes, we include the same information for another subject in inline Supplementary Figure S7.

#### Mode-specific metrics do not appear representative or stable across runs

We further defined mode-specific metrics by considering only the subsets of brain areas shifted in phase in each spatial mode, and compared their values across the 4 runs. Mode-specific metrics are commonly used to investigate differences between normal and abnormal functional brain activity (Kottaram et al., 2019; Zarghami et al., 2020). Using repeated measures ANOVA, we did not find any mode-specific metric that was reliable in all 5 modes across all 4 runs when excluding or including the cerebellar region as shown in Table 1.

**Table 1:** Repeated measures ANOVA results for mode-specific metrics over 4 fMRI acquisitions in AAL parcellations excluding the cerebellar regions (AAL90), and including the cerebellar regions (AAL116). OCC, occurrence; META, metastability; SYNC, synchronization; SPEED, typical reconfiguration speed.

Repeated measures ANOVA for mode-specific metrics				Repeated measures ANOVA for mode-specific metrics			
Significant differences				Significant differences			
AAL90				AAL116			
Metric	Mode	F score	p value	Metric	Mode	F score	p value
OCC	$\psi_{2}$	F(3294) = 10.570	*p* < 0.001	OCC	$\psi_{1}$	F(3294) = 7.158	*p* < 0.001
OCC	$\psi_{4}$	F(3294) = 5.362	*p* = 0.001	OCC	$\psi_{2}$	F(3294) = 9.634	*p* = 0.001
DURATION	$\psi_{1}$	F(3294) = 6.139	*p* = 0.001	OCC	$\psi_{3}$	F(3294) = 2.817	*p* = 0.039
DURATION	$\psi_{2}$	F(3294) = 3.152	*p* = 0.025	OCC	$\psi_{4}$	F(3294) = 8.234	*p* < 0.001
META	$\psi_{5}$	F(3294) = 7.462	*p* < 0.001	DURATION	$\psi_{1}$	F(3294) = 7.932	*p* < 0.001
SYNC	$\psi_{1}$	F(3294) = 14.466	*p* < 0.001	META	$\psi_{3}$	F(3294) = 4.262	*p* = 0.006
SYNC	$\psi_{2}$	F(3294) = 7.062	*p* < 0.001	SYNC	$\psi_{1}$	F(3294) = 11.334	*p* < 0.001
SYNC	$\psi_{3}$	F(3294) = 9.381	*p* < 0.001	SYNC	$\psi_{2}$	F(3294) = 5.138	*p* = 0.002
SYNC	$\psi_{4}$	F(3294) = 24.355	*p* < 0.001	SYNC	$\psi_{3}$	F(3294) = 3.537	*p* = 0.015
SYNC	$\psi_{5}$	F(3294) = 6.956	*p* < 0.001	SYNC	$\psi_{4}$	F(3294) = 17.132	*p* < 0.001
SPEED	$\psi_{1}$	F(3294) = 9.069	*p* < 0.001	SYNC	$\psi_{5}$	F(3294) = 85.632	*p* < 0.001
SPEED	$\psi_{2}$	F(3294) = 12.065	*p* < 0.001	SPEED	$\psi_{1}$	F(3294) = 8.552	*p* < 0.001
SPEED	$\psi_{3}$	F(3294) = 9.251	*p* < 0.001	SPEED	$\psi_{2}$	F(3294) = 16.348	*p* < 0.001
SPEED	$\psi_{4}$	F(3294) = 8.907	*p* < 0.001	SPEED	$\psi_{3}$	F(3294) = 3.272	*p* = 0.022
SPEED	$\psi_{5}$	F(3294) = 10.805	*p* < 0.001	SPEED	$\psi_{4}$	F(3294) = 17.662	*p* < 0.001
				SPEED	$\psi_{5}$	F(3294) = 65.839	*p* < 0.001

We also used a non-parametric permutation-based paired *t*-test to investigate if these differences were due to 1 idiosyncratic run or if the differences emerged over different runs. The differences remained statistically significant and were present across different runs even after performing Bonferroni correction for multiple comparisons across the 5 modes, as can be seen in inline Supplementary Figure S8. Notably, metrics of fractional occurrence and duration of modes - which have been used for comparisons between conditions studies - were not reliable across 4 acquisitions in the AAL parcellation with or without the cerebellar regions.

### Characterization of the dFC process

#### Reconfiguration speeds in phase-locking space exhibit fractal scaling and deviate from Gaussianity

An unanswered question regarding dFC is whether spatiotemporal patterns change in a discrete or continuous manner over time. K-means clustering yields a distinct mode for each timepoint, but this mode is just the cluster centroid with the shortest distance, and a number of other modes may also contribute to the resulting spatiotemporal pattern at each timepoint. An alternative perspective is to view dFC as a smooth reconfiguration of phase-locking connectivity, and to collapse these relations to a point in the space of possible relations. We can then view the evolution of this point as a stochastic exploration of a high-dimensional space. This is a direct adaptation of the reconfiguration speed introduced in (Battaglia et al., 2020) for phase-locking functional connectivity.

**Fig. 6:**
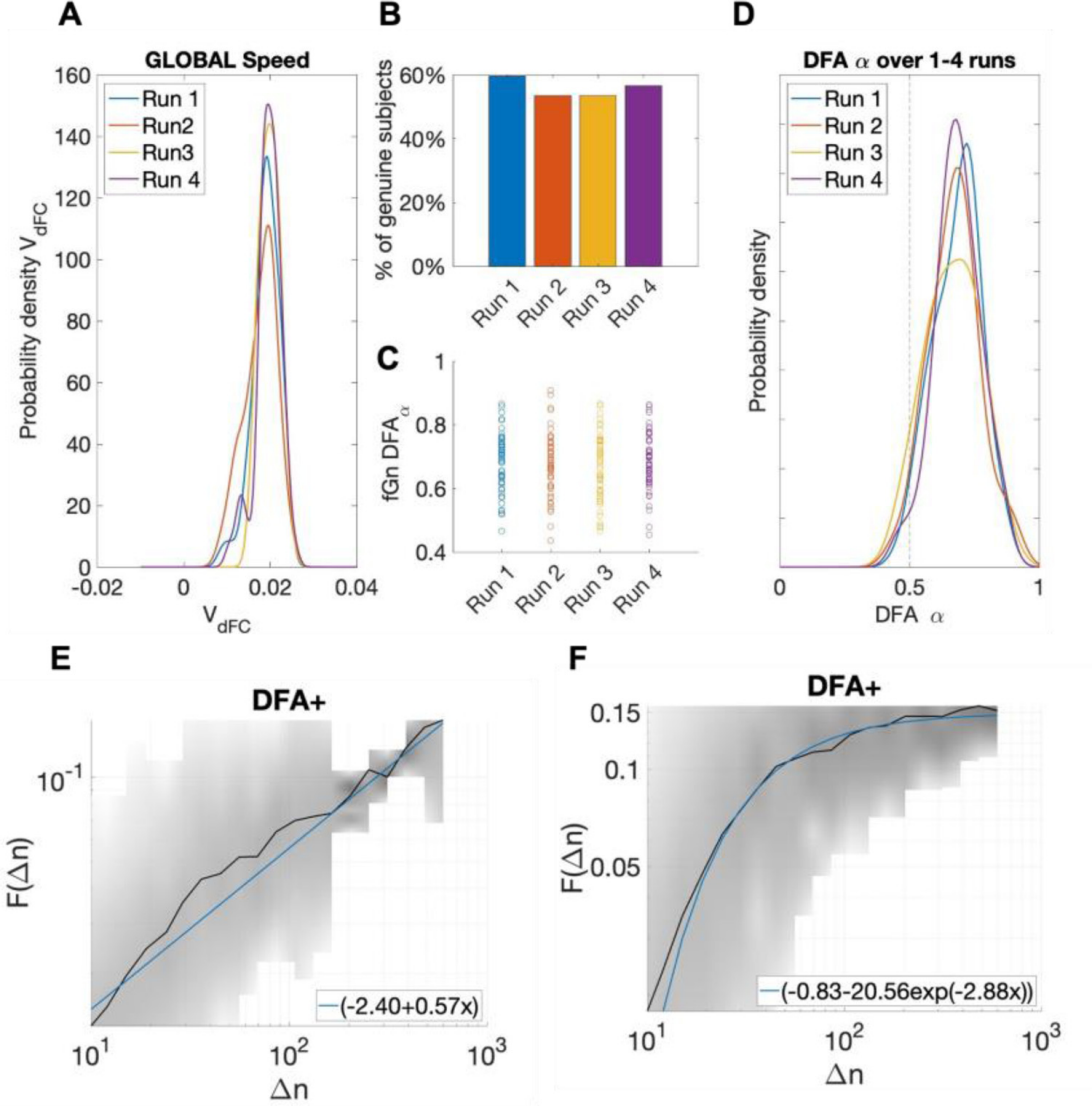
dFC reconfiguration speeds and Detrended Fluctuation Analysis (DFA). (A) Phase-coupling dFC reconfiguration speeds were slow across all 4 fMRI acquisitions. (B) Before performing DFA we established if subjects exhibited extended sections of linear power-law scaling in FC fluctuations. Between 50% to 60% of subjects exhibited ‘genuine’ power-law scaling. (C) For the majority of subjects that demonstrated linear power-law scaling, *DFA_α_* was greater than 0.5 which implies the presence of persistent fluctuations, long-range correlations, and deviation from a Gaussian generation process.  (D) The probability densities for *DFA_α_* for ‘genuine’ subjects in each of the 4 runs. (E) An example of linear power-law scaling where the best fit was found to be linear. (F) An example of non-linear power-law scaling. We used FluctuationAnalysis() (Ton and Daffertshofer, 2016) to test for linearity and to calculate *DFA*.

We computed the reconfiguration speeds (Fig. 6A) and fractal scaling characteristics (Fig. 6D) of phase-locking dFC. Our initial plan was to include the Hurst-like exponent α derived from detrended fluctuation analysis (DFA_α_) (Peng et al., 1993) in our battery of dFC metrics (see Materials and Methods). However, we found that the assumption of extended linear power-law scaling was violated in 40–50% of subjects Fig. 6 (B-C). When linear power-law scaling was present, FC fluctuations showed fractal scaling with DFA_α_ > 0.5 indicating that the stochastic reconfiguration process in phase-locking space was not random, but displayed long-range correlations and deviated from Gaussianity as shown in Fig. 6D.

**Fig. 7:**
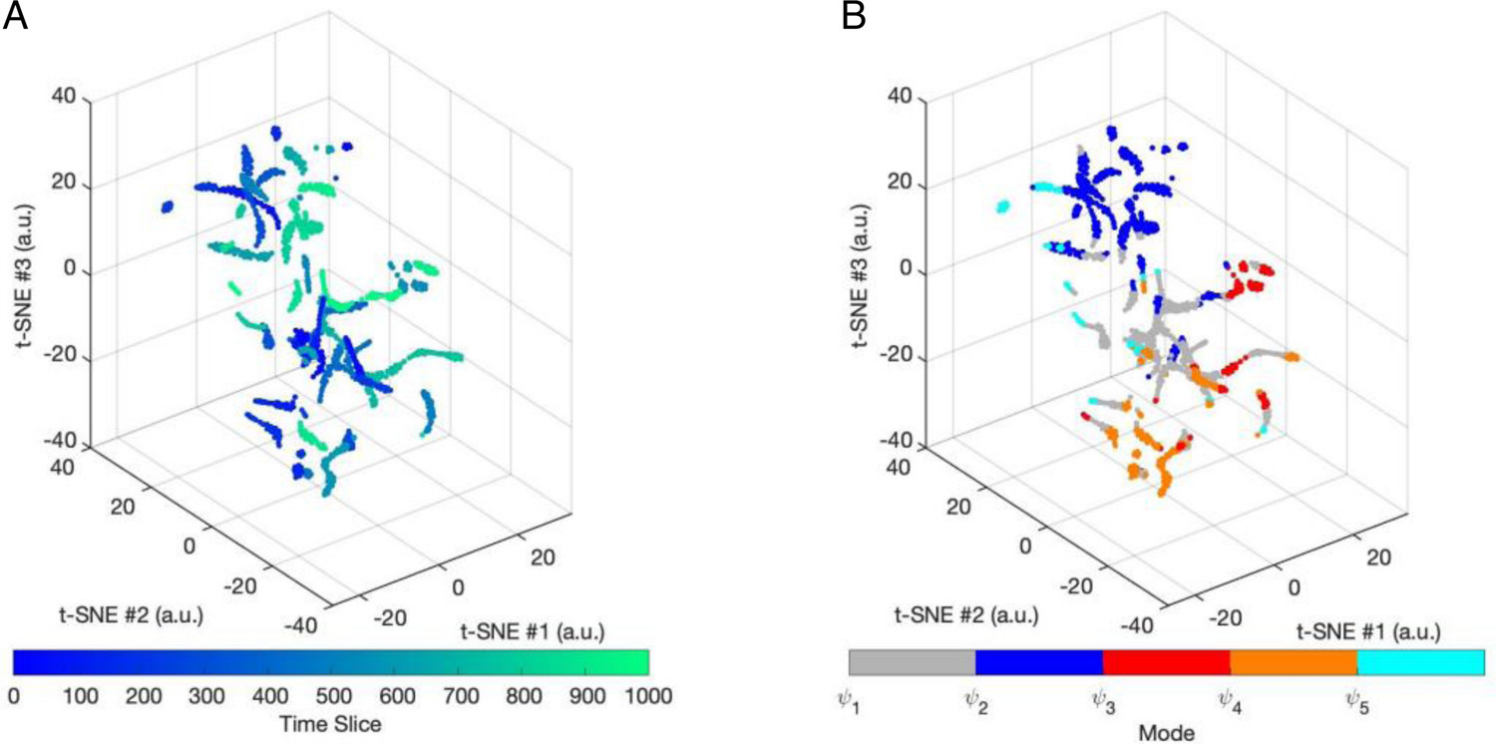
Visualizations of a reconfiguration walk in the phase space of leading eigenvectors. (A) We show a distance preserving non-linear projection in three dimensions of a subject's *dPL* stream from a single fMRI scan obtained with the t-SNE algorithm. Each point corresponds to a specific observation of FC(t) and the path connecting the points indicates in (A) the temporal order in which the different configurations are visited. (B) The same projection but color-coded with the mode assigned to the timepoint in the timeseries.

The reconfiguration random-walk of dynamic phase-locking matrices, or *dPL stream*, is represented in 3 dimensions in Fig. 7 (using a t-Stochastic Neighbor Embedding algorithm, see Materials and Methods). The resulting distance preserving non-linear projections in 3 dimensions of the associated *dPL stream* (timeseries) are shown with respect to time (left) and with respect to the mode visited (right). The speeds of reconfiguration revealed periods of slow morphing interspersed with sharp changes in the configuration of phase-locked connectivity corresponding to the concept of ‘knots and leaps’ in (Battaglia et al., 2020), in contrast to unstructured space filling.

Overall, these findings are consistent with previous literature (Battaglia et al., 2020) and suggest that spatiotemporal patterns of phase-locking change in a non-random slow continuous fashion over time.

### Relationship between global metrics

**Fig. 8:**
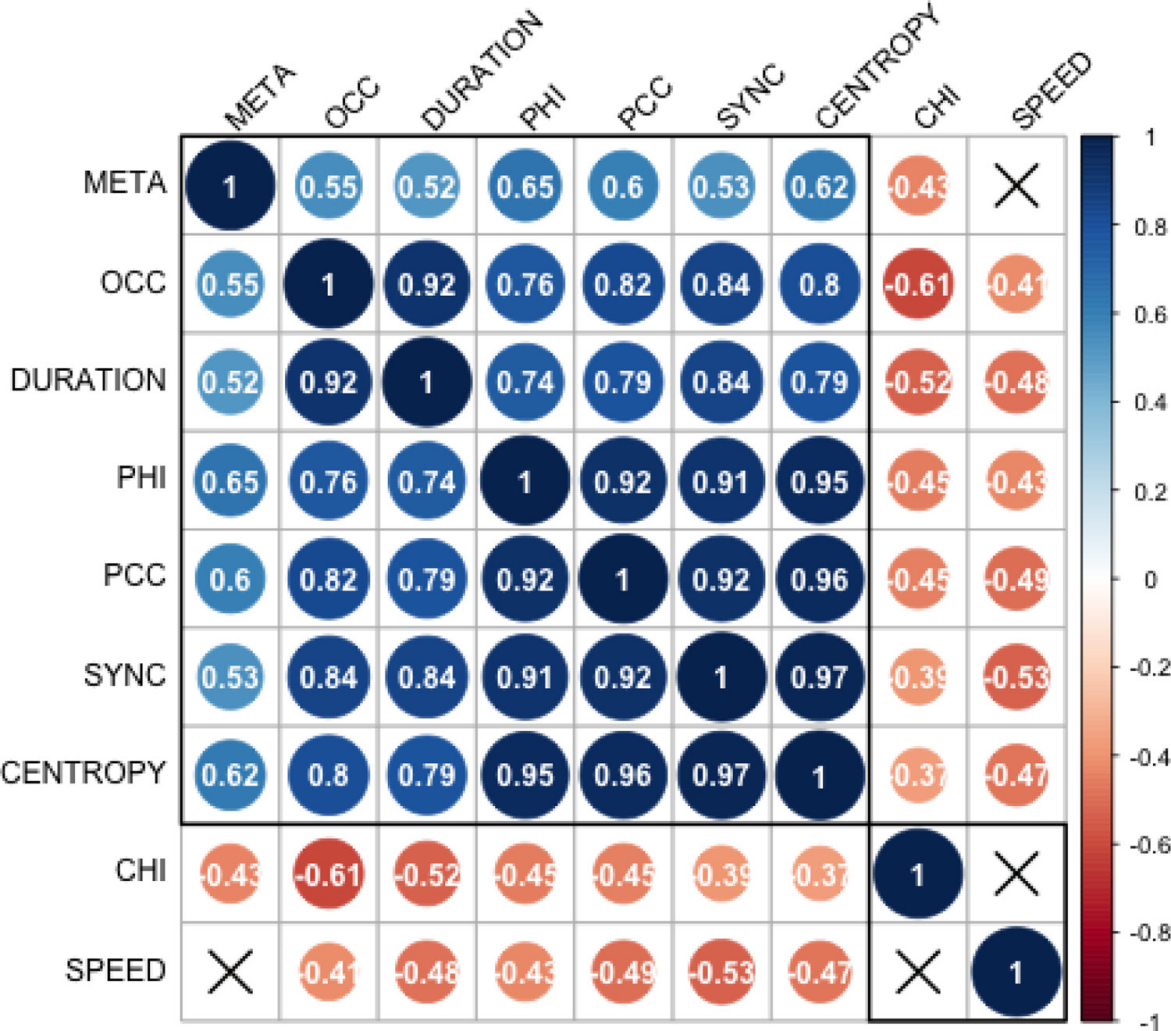
Relationships between metrics. Correlation coefficients for all metrics in run 1. Coefficients with X indicate statistical significance with α < 0.05. SYNC, synchronization; CHI, chimera index; META, metastability; OCC, fractional occurrence of $\psi_{1}$; DURATION, duration of $\psi_{1}$; SPEED, typical reconfiguration speed; PCC, phase-coherence coefficient; CENTROPY, coalition entropy; PHI, integrated information.

As a next step in our investigation, we sought to investigate how the various studied global metrics are related to each other between subjects. For this, we calculated the Spearman correlation between all pairs of metrics. As illustrated in Fig. 8 (which corresponds to RUN1), most metrics were significantly correlated, with some metrics correlating more than 90% - revealing relationships that can be more or less evident given their nature. For instance, it is not surprising that synchrony is highly correlated (*r* = 0.84) with the occupancy of the mode 1, since the latter represents more time in a mode of global phase coherence, while being also highly correlated (*r* = 0.92) with the phase coherence coefficient. Moreover, the occupancy and duration of mode 1 are also highly correlated (*r* = 0.92), which can be explained by the fact that the more a mode occurs, the more probable it is to be detected on 2 consecutive time points. Less obvious, perhaps, are the strong correlations detected between Phase Coherence Coefficient, Coalition Entropy and Integrated Information. Moreover, both the Chimera Index (CHI) and the reconfiguration speed (SPEED) exhibit negative relationships with the other metrics, but the two are not correlated to each other, indicating that they are sensitive to complementary dynamical features of the system. Correlation matrices for runs 2–4 may be found in inline Supplementary Figure S9.

To further investigate the relationship between integrated information and all other metrics, we fitted a linear mixed-effect model to predict PHI based on the values of SYNC, CENTROPY, and CHI with standardized metric values. As there appeared to be quadratic structure in the distribution of the residuals, we investigated each predictor variable in its quadratic form. SYNC^2^ provided the best model fit and so was retained as a quadratic term. Additionally, the model included random intercepts to account for the effect of different fMRI runs. The model's explanatory power related to the fixed effects alone (i.e., its marginal R^2^) is 0.85. All predictors in this model were found to be significant, with SYNC and CENTROPY displaying positive effects while CENTROPY displayed a negative effect as illustrated in Table 2.

**Table 2:** Linear mixed-effect regression model - fixed effects.

Predictor	Beta	T score	p value
SYNC^2^	0.05	t(390) = 3.03	*p* = 0.003
CENTROPY	0.87	t(390) = 42.35	*p* < 0.001
CHI	−0.11	t(390) = −5.17	*p* < 0.001

The quality of the model fit was assessed using *performance* (Lüdecke et al., 2021) and a visualization of the model checks can be found in inline Supplementary Figure S10. Additionally, this linear mixed regression model indicated that there were no random effects due to RUN as the standard deviation of the random intercept was 1.454e-16.

These findings provide unique empirical evidence that dynamical and informational complexity are related; and show convergence of evidence from multiple approaches to support the interpretability of these metrics within neuroscience.

## Discussion

When conceptualizing the brain as a complex system (Turkheimer et al., 2021) one has a number of theoretical approaches and corresponding methodological tools available to assess dynamic functional connectivity beyond viewing them as mere time-varying temporal correlations in fMRI signals. In this work we empirically investigated the relationships between approaches that investigate intrinsic brain activity from a dynamical systems perspective, from a stochastic process view, and from an information-processing perspective, providing some practical first steps towards the development of unified accounts of brain function.

Four main insights can be derived from our results. First, from a methodological perspective, phase-locking functional connectivity derived with LEiDA, provides an invariant basis of spatial modes for the investigation of dynamical behavior between brain regions. This invariant basis could be used as a template for future studies providing a validated (in terms of test-retest reliability) basis for cross study comparisons. These 5 reliable spatiotemporal modes of phase-locking activity reflect the physics of self-organization (Haken, 1996): that is, these macroscopic patterned modes are spontaneously created and change dramatically at critical points, showing how global order can emerge from local interactions (Kelso, 1995).

The patterns detected in this study closely align with those detected in previous published works applying the same LEiDA methods to datasets in different conditions (Cabral et al., 2017; Farinha et al., 2022; Figueroa et al., 2019; Lord et al., 2019), and more generally to canonical resting-state networks or intrinsic connectivity networks, suggesting these are expressions of the same phenomenology.  Although the patterns are found to diverge slightly depending on the parcellation, on the inclusion of subcortical and/or cerebellar areas, on the preprocessing steps, or on particular characteristics of the cohort, there seems to be a strong overlap across studies. In addition, we have unpublished results showing how these patterns of PL depend on the MR acquisition sequences (including multiband and in-plane acceleration), showing that some sequences may be more sensitive to some patterns than others (work in preparation).

Second, global metastability was the only representative and stable metric across a cohort of healthy young adults when the cerebellum is considered in conjunction with the cortex and subcortex. This may seem surprising given that the modes themselves were invariant across scanning sessions. However, the modes reflect centroids derived from K-means clustering, and as such represent the center of the cluster. Any particular instance or realization of a fMRI timeseries will not necessarily reflect these centroids, but will nevertheless have their time-points assigned to the mode they are closest to. The disadvantage of such hard clustering is that each time-point will only be assigned to one mode when in fact, the spatiotemporal pattern of the time-point may closely match more than one mode.

In addition to methodological considerations, there may be physiological effects that affect brain activity across runs. Indeed, within-individual changes in resting-state dynamics have been associated with fluctuations in arousal (Laumann et al., 2017), physiological state (Chang et al., 2013; Schneider et al., 2016), ongoing conscious experience (Gonzalez-Castillo et al., 2021) and spontaneous memory replay (Tambini and Davachi, 2019). Systematic differences have also been found with time of day (Orban et al., 2020; Vaisvilaite et al., 2021). However, our results indicate that global metastability is relatively insensitive to these effects. This global metric is therefore a potential candidate for neurological markers of effect in intervention studies.

Indeed, empirical results have shown global metastability to be higher when the brain was at rest (Hellyer et al., 2014), reduced during states of unconsciousness (Jobst et al., 2017), and increased beyond the resting-state maximum when the brain was in a psychedelic state (Carhart-Harris et al., 2014; Lord et al., 2019). In clinical populations, global metastability was found to be progressively reduced for mild cognitive impairment to Alzheimer's disease (Córdova-Palomera et al., 2017) and positively correlated with cognitive flexibility (Hellyer et al., 2015). Metastable synchronization of brain subsystems has also been shown to drive the transient emergence of cluster synchronization, replicating features of resting-state magnetoencephalography MEG (Cabral et al., 2014). Global metastability, is therefore, a reliable dFC metric that has promise for both empirical and computational studies.

However, as the majority of metrics were not representative across the same subjects in different acquisitions, they may not be representative or generalizable to the overall population of healthy young adults. This nonergodicity challenges the interpretation of cross-sectional study outcomes and questions the applicability of such designs to study phenomena that may be more suitable to investigation of individual life-trajectories through approaches such as fingerprinting (Van De Ville et al., 2021).

The differential effect of including the cerebellum in the calculation of our dFC metrics is intriguing. The cerebellar regions have been shown to be associated with the DMN and FPA (Buckner et al., 2011), and to be active across a range of motor and cognitive tasks including working memory, cognitive control, social cognition (King et al., 2019) and emotional processing (Pierce and Péron, 2020). Interestingly, it has been suggested that the cerebellar regions fine-tune limbic-induced synchronization of the cortical regions (Pierce and Péron, 2020) which is consistent with our findings that mode $\psi_{3}$ includes frontal-parietal, limbic, and cerebellar regions. This synchronization effect of the cerebellar regions has been neglected to date in dFC studies, but appears to play a key role for the reliability of global metastability.

Third, we sought to find reproducible evidence of convergence from multiple methods by investigating the relationship between our diversely derived metrics. The development of a prediction model that was independent of run and included metrics derived from dynamical systems theory, information theory, and information dynamics testifies to the neuroscientific interpretability of our results. It also revealed in empirical data that dynamical and informational complexity are related, confirming previous computational study findings (Mediano et al., 2022). It is interesting to note that in our regression model, the main effect of cluster synchronization was to *reduce* mean integrated information $\Phi^{R}$. What this suggests is that excessive competition between the communities to create coalitions may lead to predominantly redundant information processing; conversely, the diversity of cluster coalitions would be what leads to transfer and synergistic information processing. Integrated information - as computed in this study, with the additional subtleties of decomposition and multivariate sources and targets, may be capturing some elements of conscious processing. Intriguingly, integrated information was not significantly predicted by metastability, although moderate positive correlations between the two metrics were found in all 4 runs. Indeed, global metastability could be associated with homeostasis, reflecting a healthy regulation of tendencies for integration and segregation. Metastability can be viewed as providing the opportunity for the system to engage in cluster synchronization resulting in segregation of the communities. This dynamic segregation feature of the system appears to be complementary to the speed of changes in FC, and the metrics sensitive to these features exhibit negative relationships with all other metrics.

Taking metrics derived from dynamical systems theory and stochastic processes yields complementary insights into the dynamical complexity of brain functioning. However, not all metrics revealed findings consistent with previous literature. It is not unexpected to find periods of high phase coherence across communities in the global mode, but it would be expected to find CTC-like channels of communication when other modes are dominant. It may be that the synchronization threshold λ = 0.8 was too high to allow for delays in phase synchronization between remote communities. Indeed, when the threshold was set to λ = 0.7, periods of high phase coherence across communities were also found in other modes as can been seen in inline Supplementary Figure S11.

Computational models play a crucial role in neuroscience either for predicting phenomenon or for replicating phenomenon observed in empirical data. In this study we included metrics from both empirical data and computational modeling, and have unveiled relationships that will require a fundamental review of the underlying theoretical and mathematical concepts for neuroscientific interpretation.

Fourth, and finally, from describing brain behavior from the perspective of a stochastic process, we have provided tentative confirmatory results that the dFC process changes in a slow, non-random manner. It must be noted that we used phase-locking functional connectivity rather than temporal correlation as in the original application of this innovative methodology (Battaglia et al., 2020). Our non-linear measures of dynamic phase-locking behaved differently than linear correlations. Despite this difference, we were still able to show in the majority of subjects, that spatiotemporal patterns of phase-locking change in a continuous and non-random manner, exhibiting long-range temporal correlations, indicating the presence of memory.

Taken together, these results are congruent with complex systems theory (Turkheimer et al., 2021) in that phase-relationships in fMRI of the resting state brain exhibit:

• Invariant spatiotemporal patterns that are indicative of self-organized processes (Haken, 1996).
• Nonergodicity in that dFC metrics are in general, not representative across samples (Turkheimer et al., 2021).
• Diversity in cluster synchronization (Turkheimer et al., 2021).
• Fractal scaling in the continuous change of functional connectivity (Battaglia et al., 2020).

## Limitations and future research

A number of limitations that should be considered when evaluating the findings. Starting with modes, we found near perfect ICC agreement of all 5 spatiotemporal phase-locking modes across all 4 runs. However, ICC is a relative metric and the large between-region differences may bias a high ICC value in the absence of genuinely small within-region differences. However, we achieved similar results with Pearson correlation.

Another possible limitation of this study is that in contrast to previous studies of metastability, we defined the communities of oscillators directly from the phase-locking data and not from intrinsic connectivity networks. Our so-derived communities are not distinct, specifically mode $\psi_{1}$ comprises all other modes. This may be a violation of assumptions for calculating some metrics but we believe that it is more representative of what may actually be happening in the brain, that is, coalitions transiently forming between phase-related communities.

Moving on to communities, previous investigations of $\Phi^{R}$ in fMRI data have used a continuous model to compute the relevant information theoretic variables (Luppi et al., 2022). In this study we adopted the discrete data model which has been used in computational models of weakly coupled Kuramoto oscillators (Mediano et al., 2022, Mediano et al., 2016). We computed $\Phi^{R}$ for an integration timescale from 1 to 500 TRs and retained the max $\Phi^{R}$ obtained as indicative of integrated information for a specific subject in a specific run. Although there is information in the integration timescale that yielded this $\Phi_{max}^{R}$, a maximum statistic test (Novelli et al., 2019) would be required before any inferences may be drawn.

We note that there are a number of differences between our findings and those of (Battaglia et al., 2020). Our stochastic walks were based on instantaneous phase-locking and not on smoothed sliding-window temporal correlation. We used a parcellation with 116 rather than 68 anatomical regions which influences the resulting speeds, and potentially the power-law scaling and fluctuation characteristics. We also did not pool our data as we had sufficient datapoints (1198 TRs) for our calculations. Unlike Battaglia et al. we found that between 40 and 50% of the HCP subjects exhibited a loss of linearity in power-law scaling in any particular run. In fact, just 7 subjects showed ‘genuine’ power-law scaling over the 4 runs. In a previous study investigating fractal scaling in phase synchronization, fluctuations were averaged over all subjects before determining the scaling component α (Daffertshofer et al., 2018) potentially obscuring loss of linearity in some individual subjects. The lack of linear power-law scaling in individual subjects has been noted before (Botcharova, 2014). We did not investigate the reasons for this lack or loss of linearity although there have been suggestions that this may be due to periodic trends (Hu et al., 2001), non-stationarities (Chen et al., 2002) or non-linear transformations (Chen et al., 2005). Indeed, it has recently been reported that different RSNs exhibit different degrees of non-stationarity (Guan et al., 2020). unraveling the reasons for loss of linearity is beyond the scope of the present paper, but merits future study.

We did not develop any null models to test the validity of the methodologies employed which may be considered a weakness of this study. However, each of these methodologies has already been validated against null models or with surrogate data (Battaglia et al., 2020; Honari et al., 2021; Mediano et al., 2022). In contrast, there have been few studies that used these methodologies to compare performance across fMRI realizations.

We have just started to explore the relationships between metrics from different conceptualizations of brain functioning. It is clear that there are a number of possible avenues for future research arising from this study. An investigation into the gender specificity of the 5 invariant modes warrants further investigation. Indeed, we found that the inter-class correlation of mode 5 between female and male subjects (ICC = 0.89) was lower than the that between runs (ICC = 0.94). This indicates that there was more variance in mode 5 between genders than between runs (see inline Supplementary Figure S12). Another aspect worth investigating is the behavior of the metrics in narrow frequency bands. An initial comparison of global metastability and synchrony across 5 frequency bands between 0.01 and 0.08 Hz indicates that the behavior may indeed differ (see inline Supplementary Figure S13). A deeper investigation of power-law linearity differences between subjects and runs for reconfiguration speeds could reveal interesting trait or state correlations. Understanding the relationships between the metrics in general, and with respect to integrated information specifically, poses a challenging task. unraveling these relationships, potentially with computational models, may provide novel insight into the mechanisms and dynamics of functional connectivity. Finally, applying this battery of metrics to longitudinal or individual life-trajectories could uncover novel relationships that have evaded detection with single methodologies.

## Concluding remarks

Neuromarkers need to demonstrate reliability and interpretability before introduction into a clinical environment. A measure of global metastability, a universal phenomenon across multiple conceptualizations of intrinsic brain activity, was found to be the most representative and stable across multiple fMRI acquisitions of the same subjects. This nonergodicity challenges the use of cross-sectional study designs for dFC. Using concepts and tools from complexity science we have described the metastable behavior of fMRI resting-state activity and our findings are congruent with complex system theory. The inter-relationships between metrics derived from dynamical systems theory, information theory, and information dynamics highlight the simultaneous and balanced tendencies for functional segregation and global integration in the healthy brain. Our battery of metrics may one day help to understand why this balance is lost in psychiatric disorders, or how pharmacological interventions can affect this balance.

## Declaration of Competing Interest

The authors declare that they have no known competing financial interests or personal relationships that could have appeared to influence the work reported in this paper.

## Appendix Supplementary materials

Image, application 1
