# Supplementary material for: Metastability, fractal scaling, and synergistic information processing: What phase relationships reveal about intrinsic brain activity
Source: Neuroimage. 2022 Oct 1;259:119433. doi: 10.1016/j.neuroimage.2022.119433 (PMC9339663; doi:10.1016/j.neuroimage.2022.119433)
Supplement: Supplementary file 1 [file mmc1.pdf]

Supplementary Materials for ‘Metastability, fractal-scaling, and synergistic information processing: what phase relationships reveal about intrinsic brain activity’, *Hancock et al.* (2022)

A

Supplementary Figures (S1-S13) **2**

Supplementary Tables (ST1-ST4) **15**

B

Supplementary Methods and Metrics **18**

References **30**

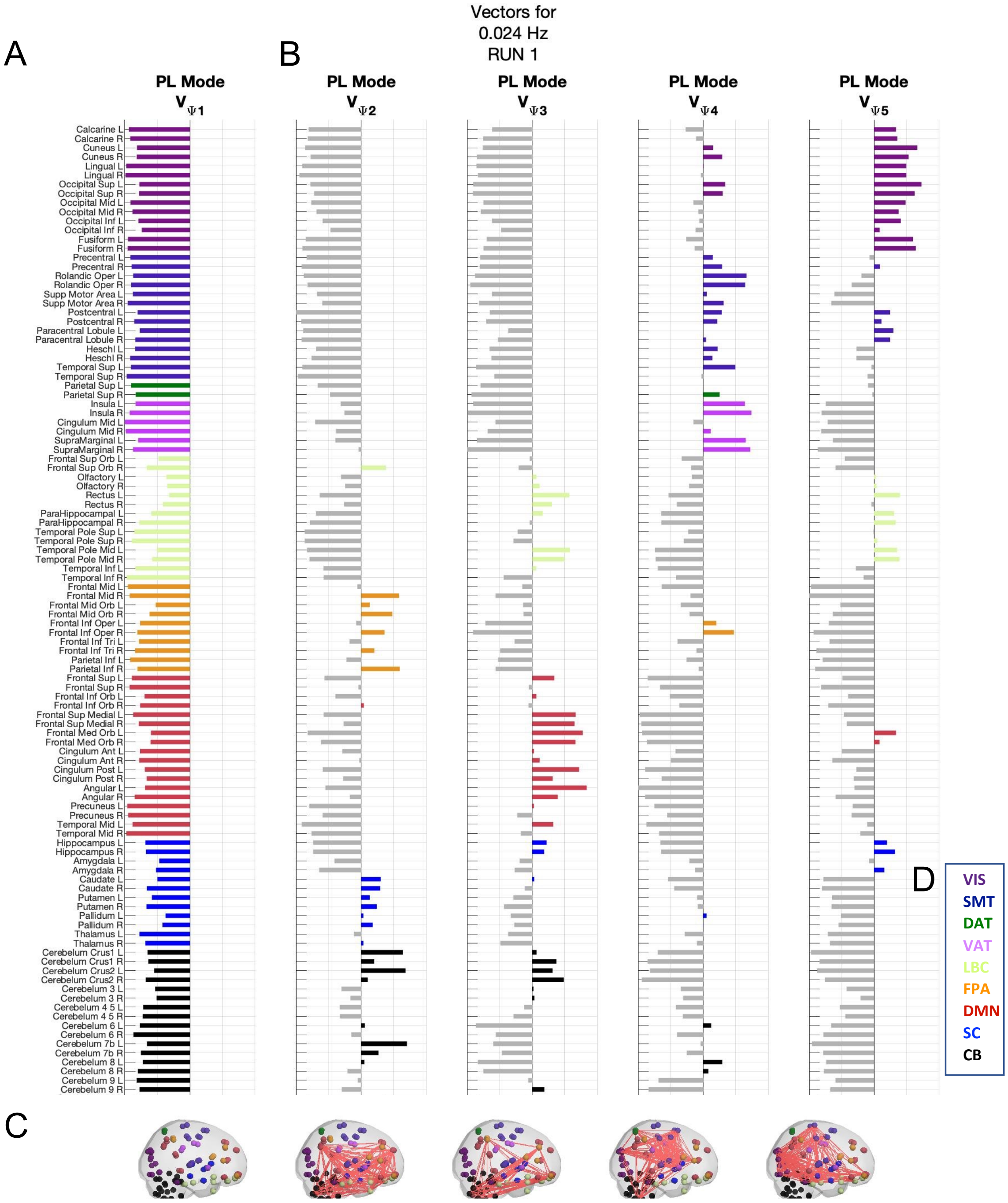

Supplementary Figure S1 Spatiotemporal patterns at low frequency narrow bands

The spatiotemporal patterns extracted between 0.010 Hz and 0.024 Hz do not resemble noise. Rather they resemble the spatiotemporal patterns extracted from the broad band 0.01 – 0.08 Hz in Supplementary Figure S5 below. (A) The bar plot shows the elements in V_c with anti-phase synchronization in mode ψ_1, the global mode. (B) The bar plots show the elements in V_c with in-phase synchronization in modes ψ_2 to ψ_5 color coded to the 7 Yeo RSNs. (C) Representation of the 5 modes obtained when including the cerebellum. We rendered the eigenvectors in cortical space by representing each element as a sphere placed at the center of gravity of the corresponding brain region, and coloring the spheres according to the assigned Yeo resting-state network. We also plot links between the corresponding areas to highlight the network formed by the smallest community of brain areas. (D) Color-codes for the YEO 7 RSNs, visual (VIS), sensory-motor (SM), dorsal attention

(DAT), ventral attention (VAT), limbic (LBC) frontal-parietal (FPA), default mode (DMN), the sub-cortical (SC) and cerebella (CB) regions.

Bedrosian – Carson's empirical rule 'useful bandwidth B_φ'

B_φ = 2(Modulation Index +1)f_m

A B

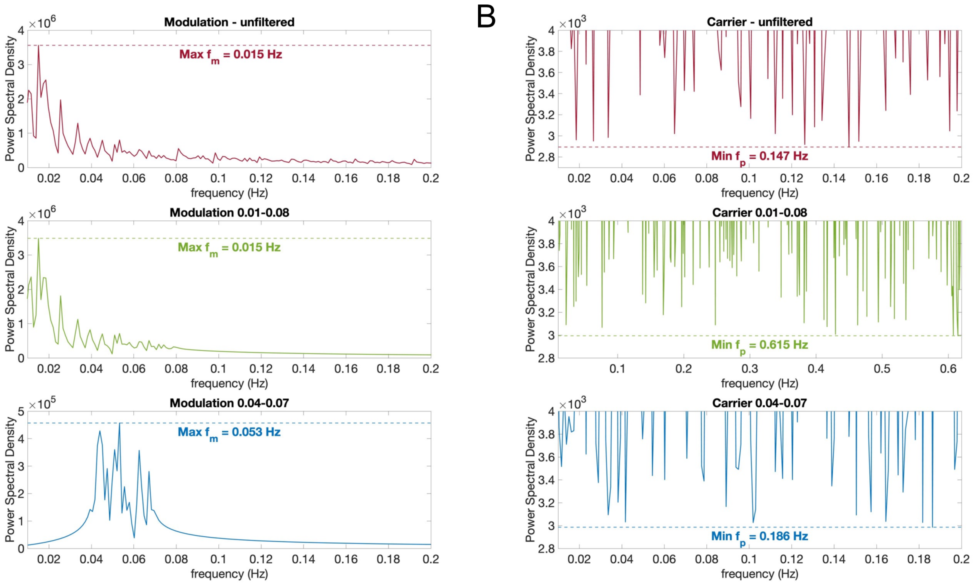

C

	Max f_m - $Freq_{Amplitude}$	Modulation Index	Useful bandwidth B_φ	Min f_p - $Freq_{Phase}$
unfiltered	0.015 Hz	0.574	0.047 Hz	0.147 Hz
0.01-0.08	0.015 Hz	0.580	0.048 Hz	0.615 Hz
0.04-0.07	0.053 Hz	0.571	0.167 Hz	0.186 Hz

D

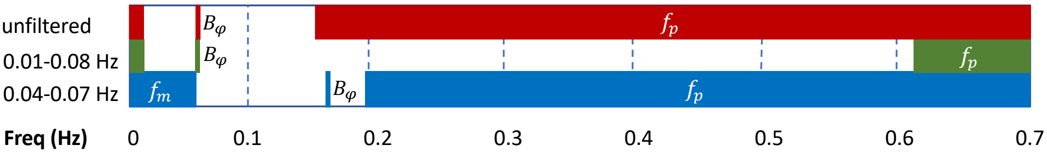

Supplementary Figure S2 Carson's empirical rule for non-violation of the Bedrosian Theorem (A) The peak frequency for the modulation signal Max f_m is calculated from the power spectrum of the unfiltered and band-passed analytical signals. This corresponds to the max frequency in the amplitude component of the analytical signal. (B) The lowest frequency for the carrier signal Min f_p is calculated from the power spectrum of the unfiltered and band-passed filtered phase component of the analytical signals. This corresponds to the lowest frequency in the phase component of the analytical signals. (C) The useful bandwidth B_φ is calculated from Carson's empirical rule. (D) The spectral support of the amplitude and phase functions are separate in both unfiltered and band-passed filtered fMRI signals in HCPU100 image data.

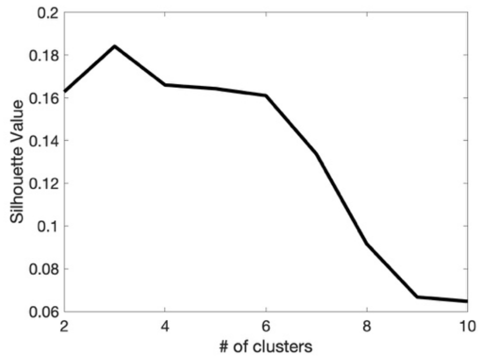

Supplementary Figure S3 Silhouette values for clustering solutions between k=2 and k=10

A

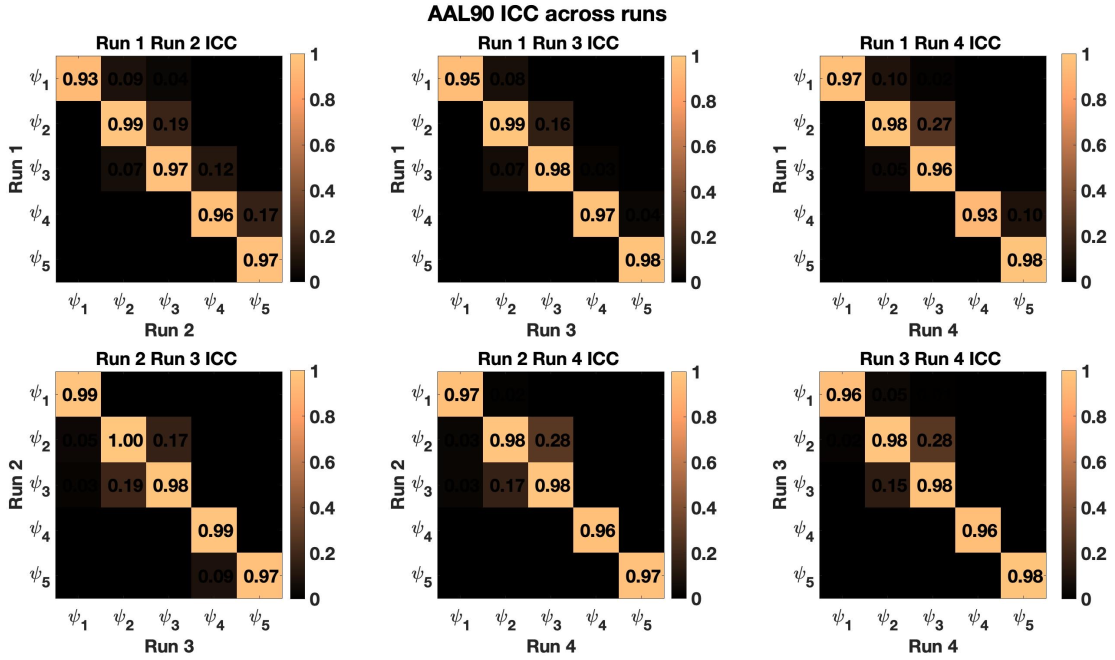

B

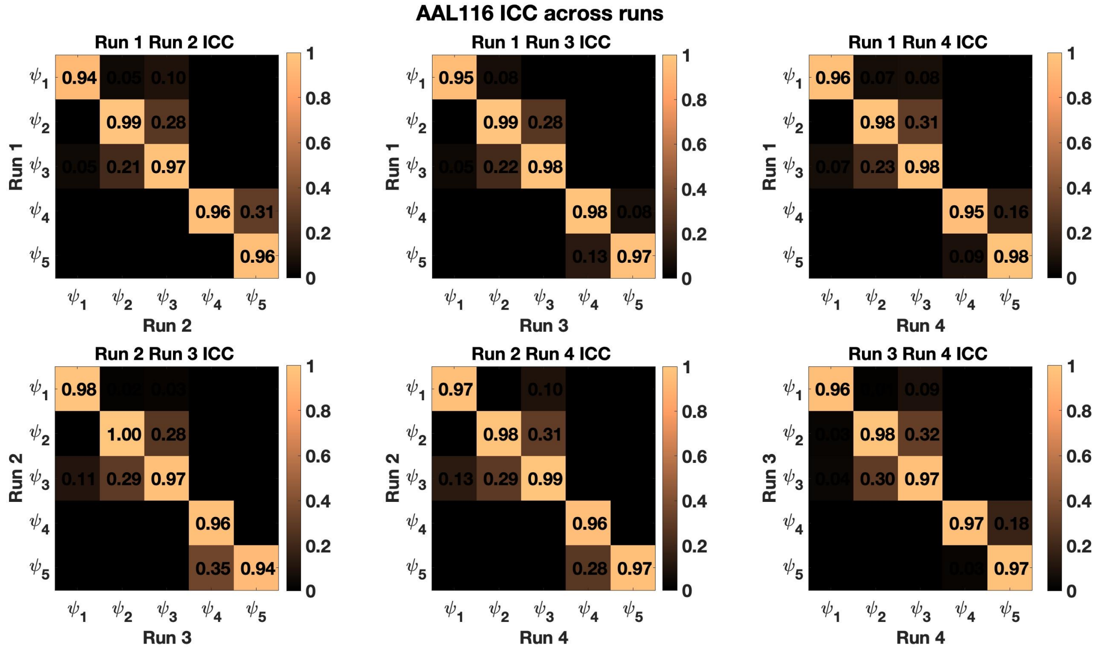

Supplementary Figure S4 Interclass correlation coefficients (ICC) for spatiotemporal patterns of phase-locking extracted with K-means clustering.

(A) Spatiotemporal modes extracted in AAL with 90 regions showed almost perfect agreement between all runs 0.97 > ICC > 0.99. (B) With the inclusion of the cerebellar regions, the order of mode extraction differed between runs. Accounting for this difference run reliability again showed almost perfect agreement 0.94 > ICC > 1.0 in AAL with 116 regions.

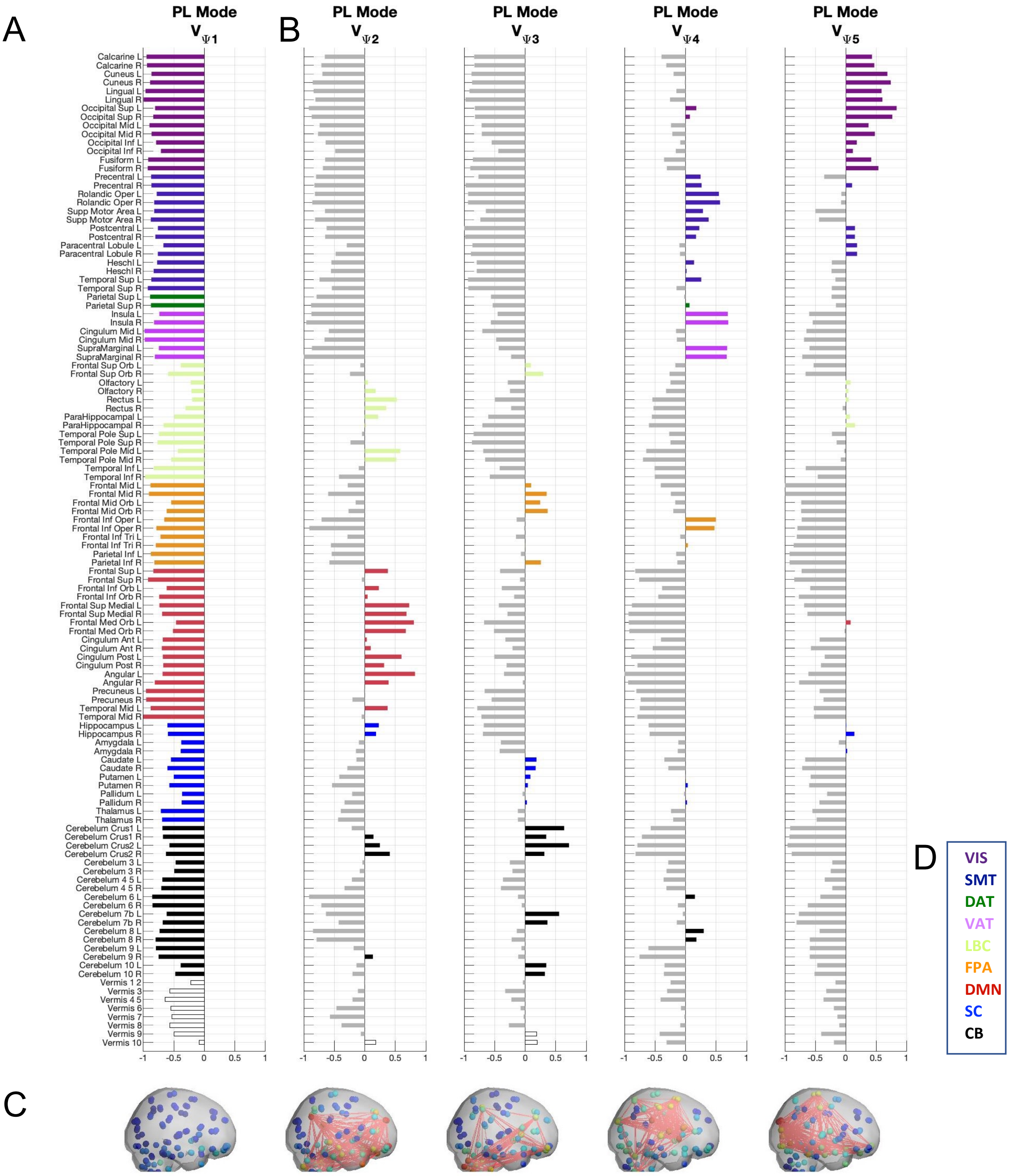

Supplementary Figure S5 Phase-locking modes include sub-elements of Yeo's 7 canonical resting state networks (RSNs).

(A) The bar plot shows the elements in V_c with anti-phase synchronization in mode ψ_1, the global mode. (B) The bar plots show the elements in V_c with in-phase synchronization in modes ψ_2 to ψ_5 color coded to the 7 Yeo RSNs. (C) Representation of the 5 modes obtained when including the cerebellum. We rendered the centroid vectors V_c in cortical space by representing each element as a sphere placed at the center of gravity of the relevant brain region, and scaling the color of the spheres according to the value of the relevant eigenvector. Regions with similar phase orientation are colored alike (yellow-to-red for the smallest community and cyan-to-blue for the largest community), where darker colors (red/blue) indicate weak contributions and lighter colors (cyan/yellow) indicate stronger contributions. (D) Color-codes for the YEO 7 RSNs, visual (VIS), sensory-motor (SM), dorsal attention

(DAT), ventral attention (VAT), limbic (LBC) frontal-parietal (FPA), default mode (DMN), the sub-cortical (SC) and cerebella (CB) regions.

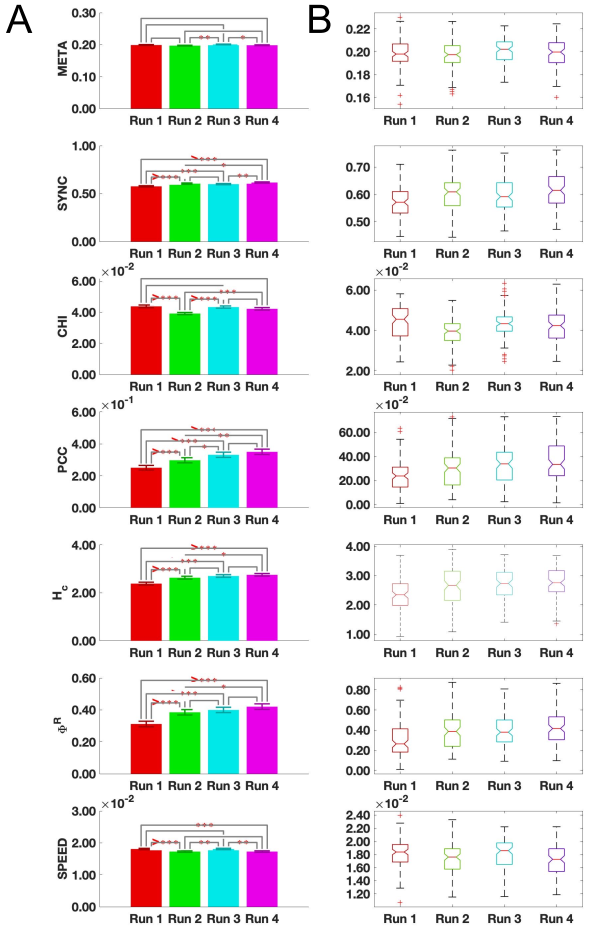

Supplementary Figure S6 Reliability of metastability in parcellations without the cerebellar regions (A) AAL90: The mean values for each metric are shown as bar plots. The *** indicate a statistically significant difference between metrics across the associated runs where * $p < 0.05$, ** $p < 0.01$, *** $p < 0.001$, and >*** $p < 0.0001$. (B) AAL90: The distribution of the global metrics across runs. META, metastability, SYNC, synchronization, CHI, chimera index, PCC, phase-coherence coefficient, H_C coalition entropy, Φ^R, integrated information, SPEED, typical reconfiguration speed.

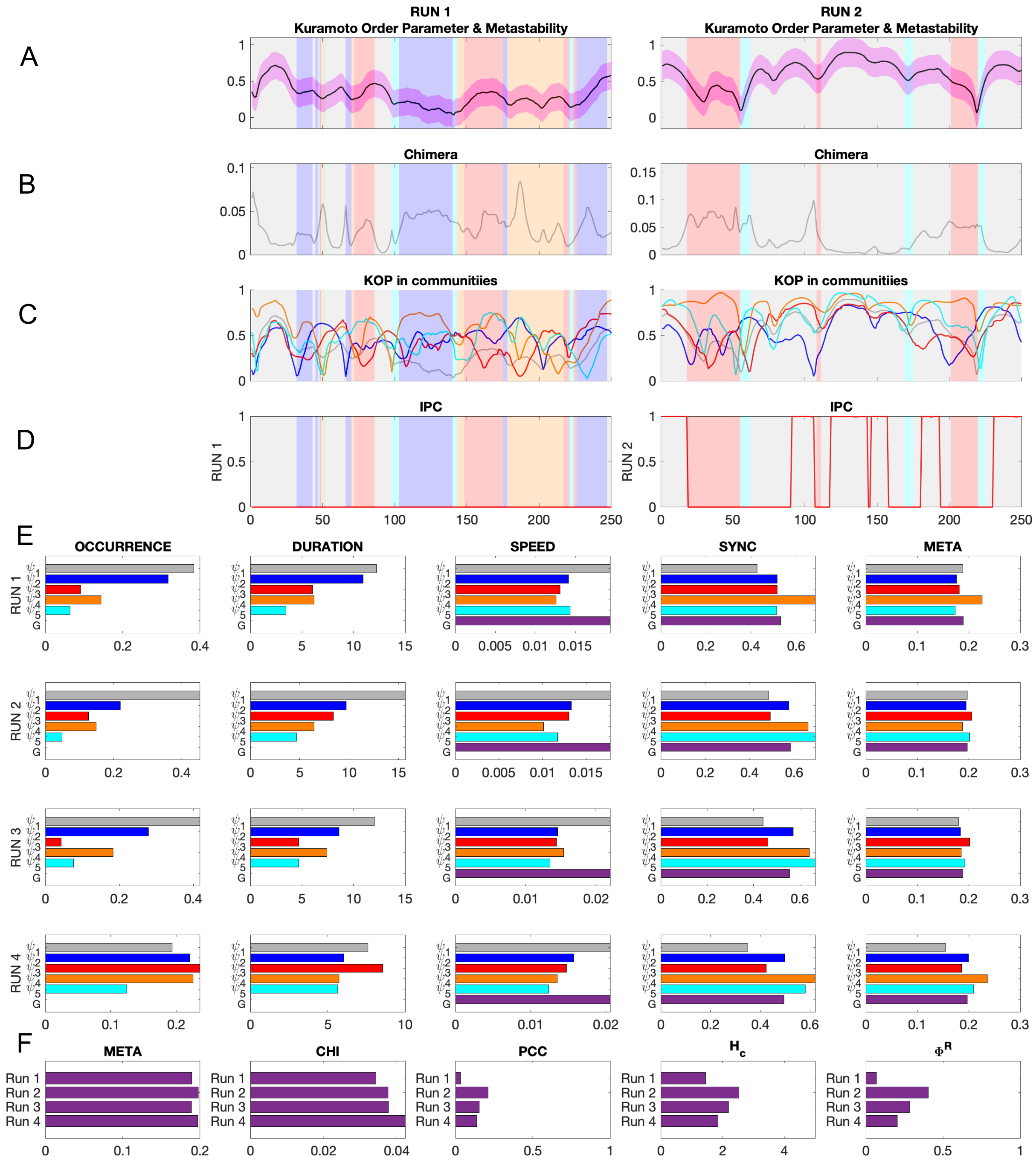

Supplementary Figure S7 Overview of all metrics in all runs for a representative subject.

(A) Exemplar snippets from the instantaneous phase synchrony or Kuramoto order parameter time series for each run color-coded to show which mode was dominant at the time. (B) The same as A but for chimeras or cluster synchronization. (C) The evolution of instantaneous synchrony in each of the color-coded modes. (D) The evolution of instantaneous phase-coherence. (E) The values of the mode-specific. (F) The values of the global metrics across all 4 runs. META, metastability, CHI, chimera index, PCC, phase coherence coefficient, H_c, coalition entropy, and Φ^R, integrated information

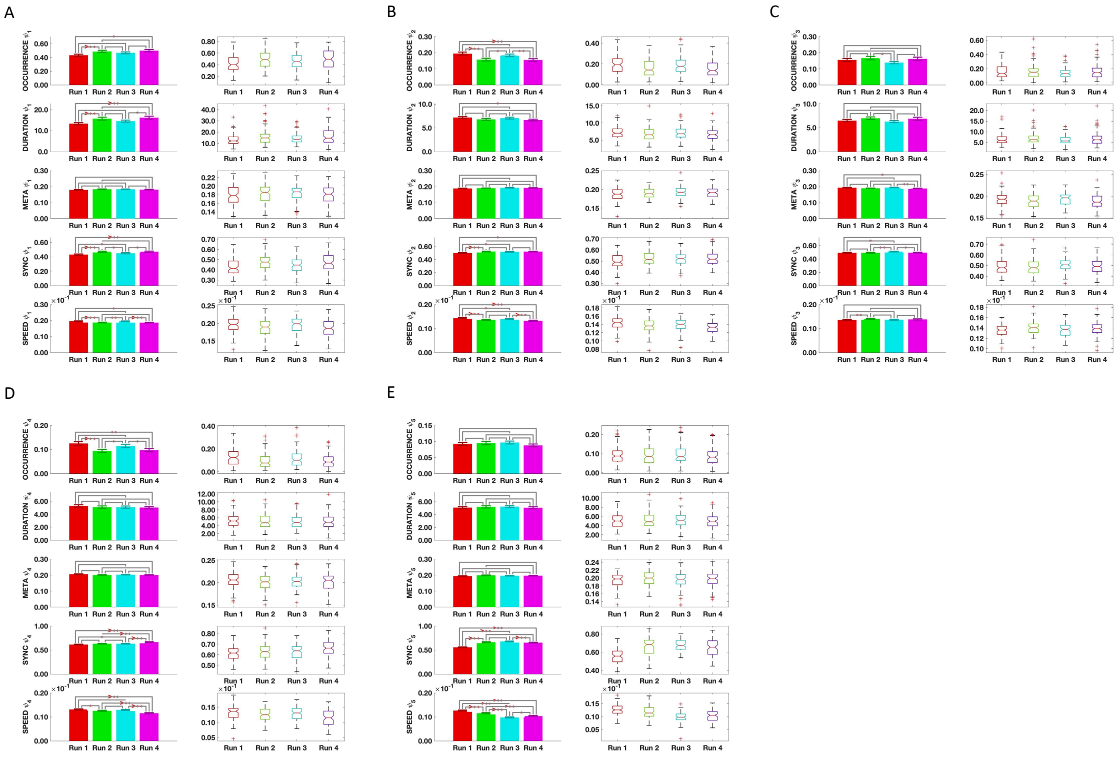

Supplementary Figure S8 Stability of mode-specific metrics in AAL116 for mode $\psi_1 - \psi_5$ across 4 runs.

(A-E) Modes $\psi_1 - \psi_5$ respectively. The mean values for each metric are shown as bar plots. The *** indicate a statistically significant difference between the metric across the associated runs where * $p < 0.01$, ** $p < 0.001$, and *** $p < 0.0001$. (B) The distribution of the global metrics across runs. META, metastability, SYNC; synchronization, SPEED, typical speed of reconfiguration.

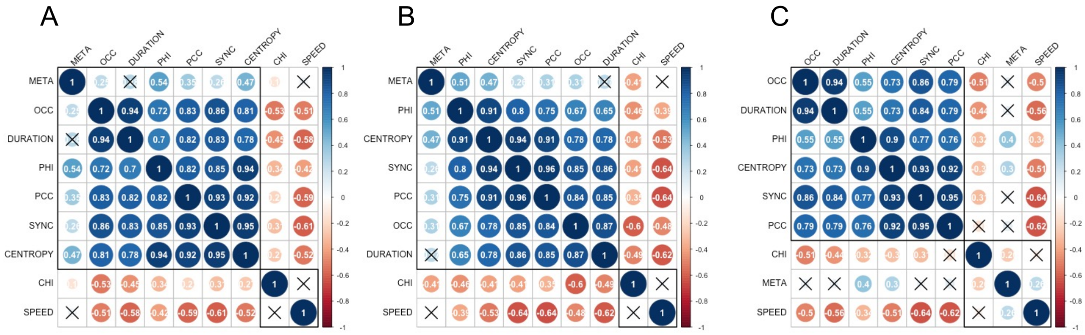

Supplementary Figure S9 Spearman correlation matrices for dFC metrics. (A) Run 2. (B) Run 3. (C) Run 4. META, metastability; OCC, occurrence; PHI, integrated information; PCC, phase coherence coefficient; SYNC, synchronization; CENTROPY, coalition entropy; CHI, chimera index; SPEED, typical reconfiguration speed.

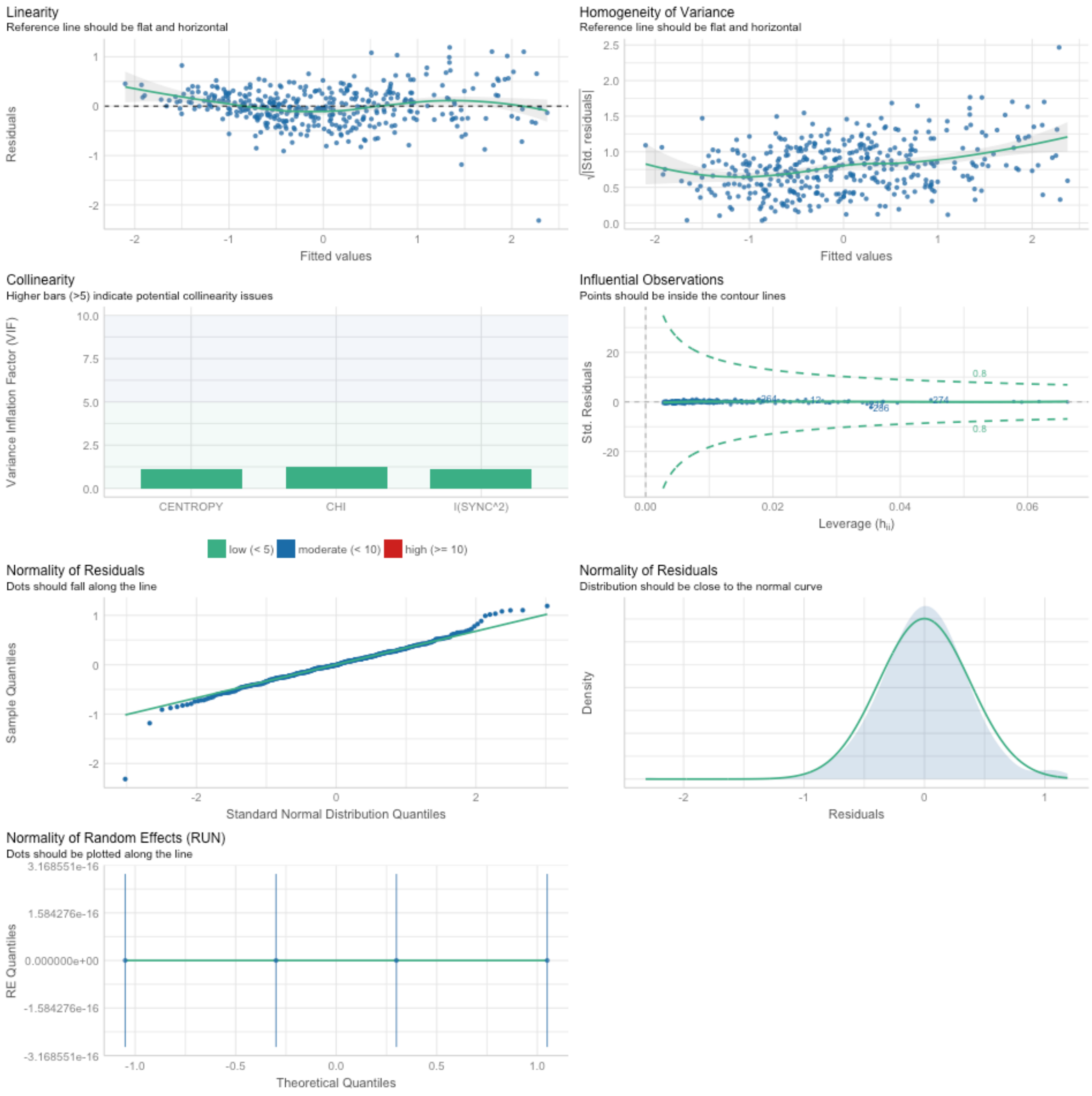

Supplementary Figure S10 Comprehensive visualization of model checks performed on the linear mixed effects regression model to predict Integrated Information from Coalition entropy, Chimera index, and Synchronization. CENTROPY, coalition entropy; CHI, chimera index; SYNC, synchronization.

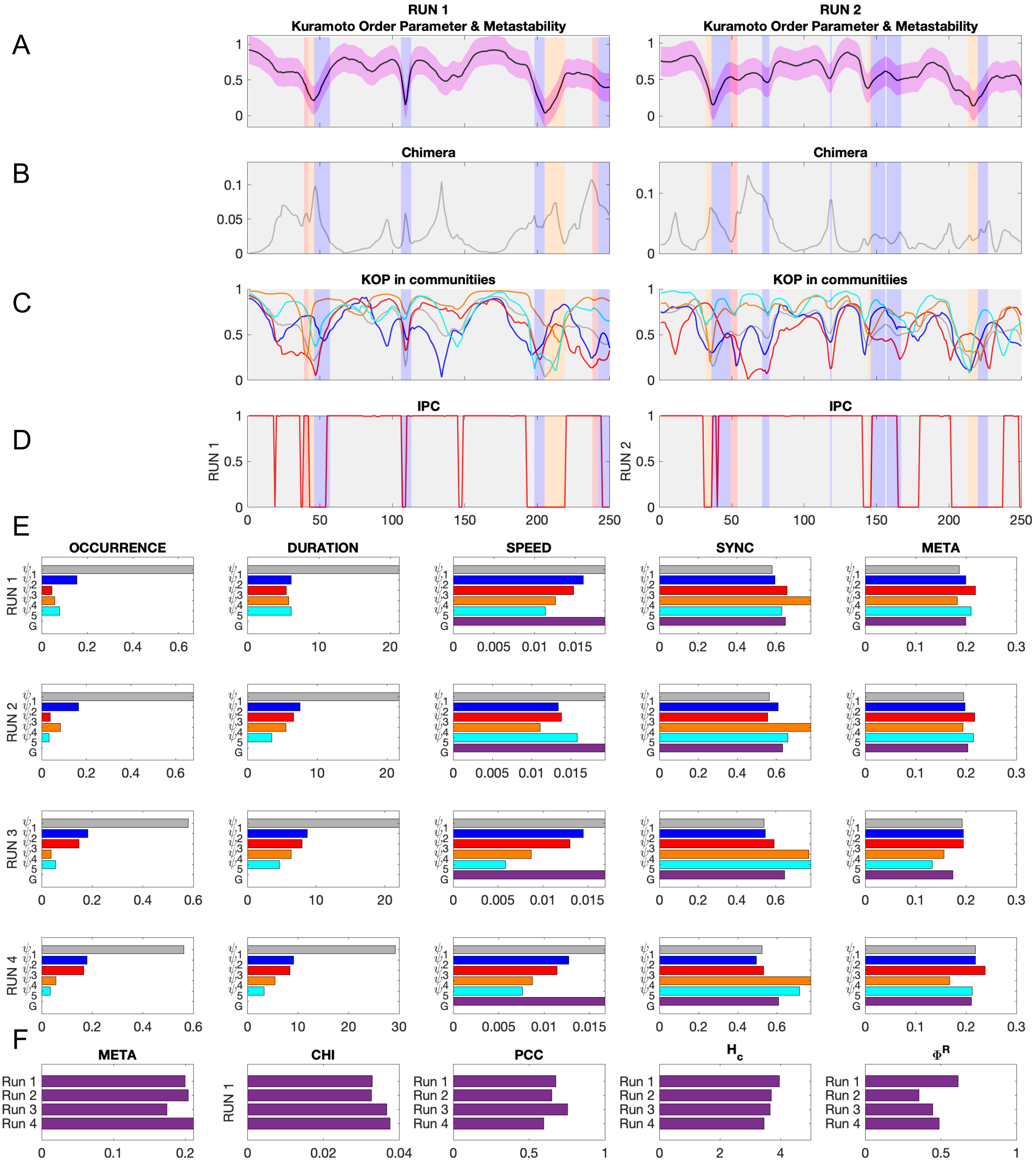

Supplementary Figure 11 Overview of all metrics in all runs for a representative subject with synchronization threshold λ = 0.7.

(A) Exemplar snippets from the instantaneous phase synchrony or Kuramoto order parameter time series for each run color-coded to show which mode was dominant at the time. (B) The same as A but for chimeras or cluster synchronization. (C) The evolution of instantaneous synchrony in each of the color-coded modes. (D) The evolution of instantaneous phase-coherence. (E) The values of the mode-specific. (F) The values of the global metrics across all 4 runs. META, metastability, CHI, chimera index, PCC, phase coherence coefficient, Hc, coalition entropy, and Φ^R, integrated information.

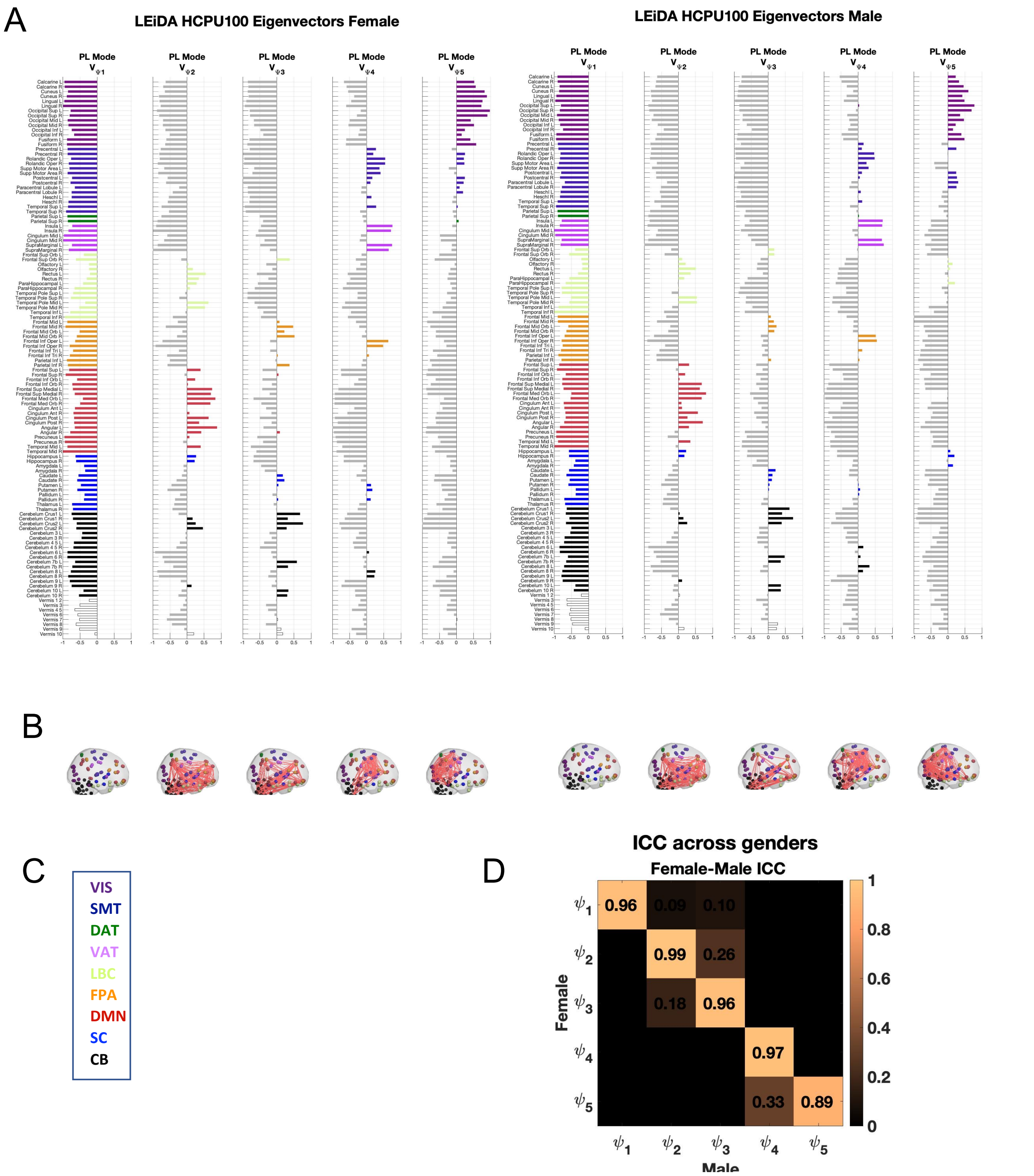

Supplementary Figure S12 Differences in phase-locking modes between female and male subjects. (A) The bar plots show the LEiDA Eigenvectors for female and male subjects. In mode 5, there is more in-phase locking of the SMT in females, and more SC in-phase locking in males. (B) Representation of the 5 modes. We rendered the eigenvectors in cortical space by representing each element as a sphere placed at the center of gravity of the corresponding brain region, and coloring the spheres according to the assigned Yeo resting-state network. We also plot links between the corresponding areas to highlight the network formed by the smallest community of brain areas. (C) Color-codes for the YEO 7 RSNs, visual (VIS), sensory-motor (SM), dorsal attention (DAT), ventral attention (VAT), limbic (LBC) frontal-parietal (FPA), default mode (DMN), the sub-cortical (SC) and cerebellar (CB) regions. (D) Inter-class correlation between females and males for 5 modes.

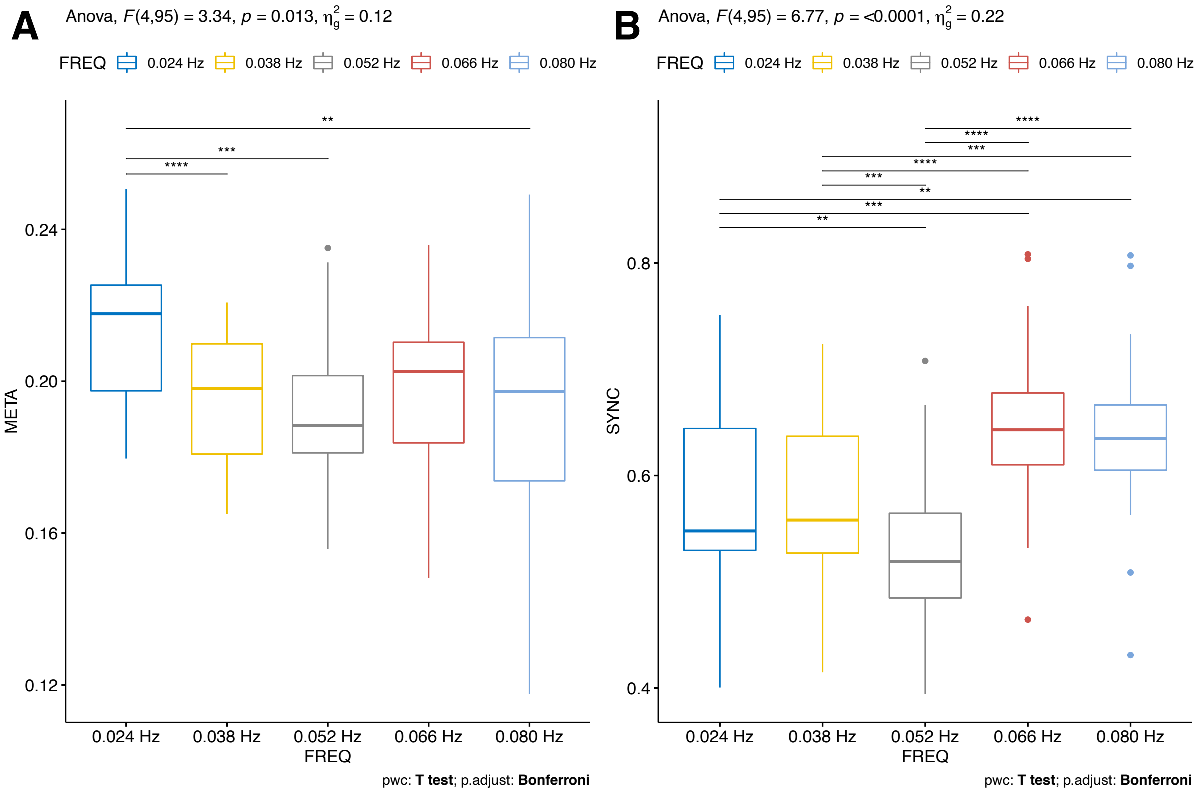

Supplementary Figure S13 ANOVA for META and SYNC across 5 frequency bands

fMRI signals were decomposed into 5 frequency bands between 0.01 Hz and 0.08 Hz using wavelet transforms. The x-axis denotes the cut-off frequency for the relevant band. For example 0.024 Hz is the high cut-off frequency for the 0.01 Hz - 0.024 Hz band.

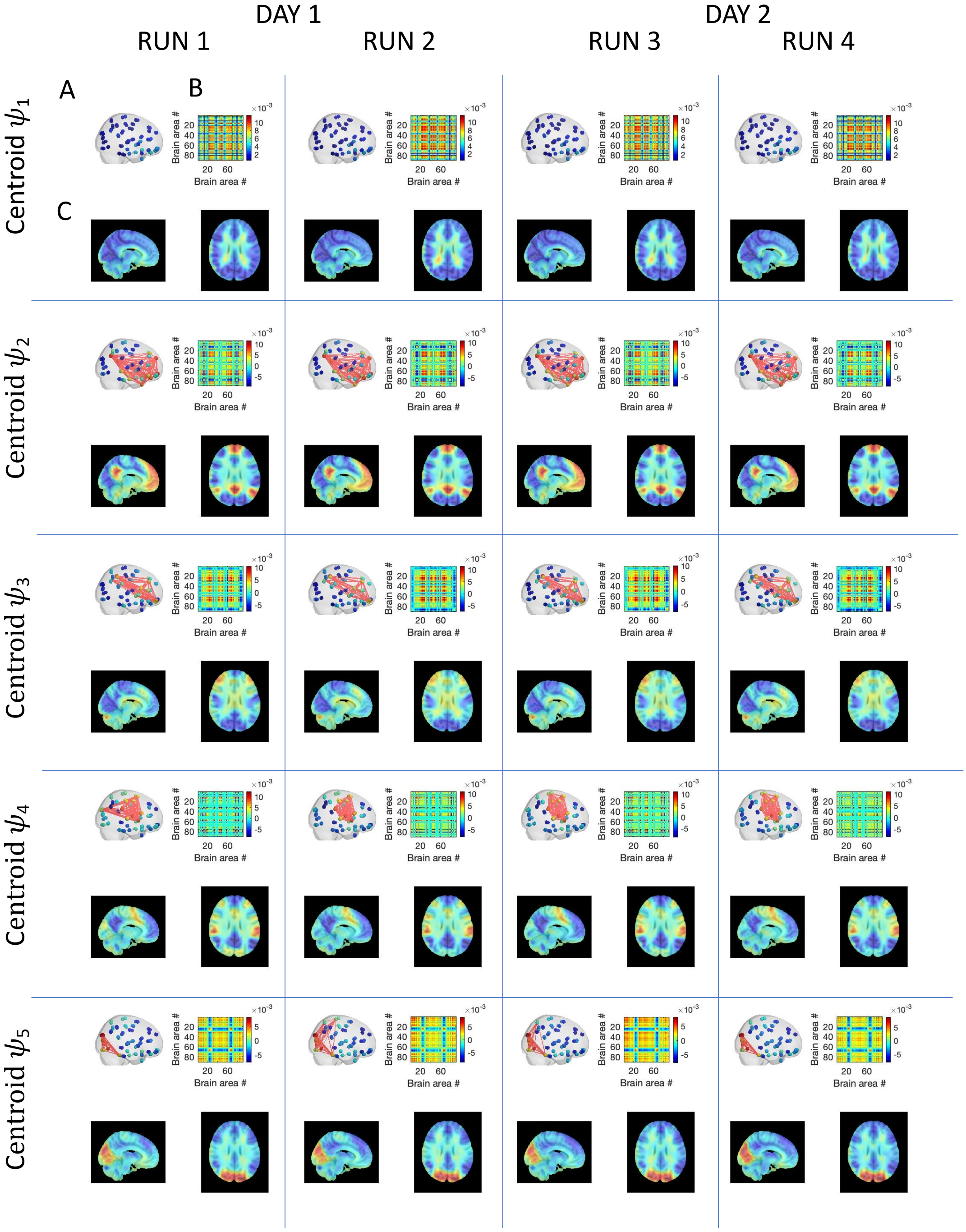

Supplementary Figure S14 Invariant spatiotemporal patterns of phase-locking across 116 regions obtained independently in each of the 4 runs on the same 99 participants.

LEiDA was applied separately to the 4 fMRI runs recorded on 2 consecutive days from 99 participants and the centroids obtained from clustering into K=5 are reported. Each centroid Vc (with size 1xN, with N=116) is represented in three distinct forms: (A) each element Vc(n) is represented as a sphere placed at the center of gravity of the corresponding brain region and its color is scaled according to its value in Vc. Links highlight the network formed by the smallest community of brain areas. (B) the phase-locking matrices computed as the outer product of the centroid vector Vc. (C) Representation of the centroid vector for each mode in 10mm3 voxel space by averaging the eigenvector values over all time instances assigned to a particular cluster/mode. The modes were then plotted over a 1mm3 MNI T1 image.

100307.' ':Day 1	20:04:46	rfMRI_REST1_RL	100307.' ':Day 1	20:19:48	rfMRI_REST1_LR	100307.' ':Day 2	17:34:04	rfMRI_REST2_RL	100307.' ':Day 2	17:49:00	rfMRI_REST2_LR
111312.' ':Day 1	11:54:45	rfMRI_REST1_RL	111312.' ':Day 1	12:10:09	rfMRI_REST1_LR	111312.' ':Day 2	09:09:02	rfMRI_REST2_RL	111312.' ':Day 2	09:24:11	rfMRI_REST2_LR
672756.' ':Day 1	18:20:30	rfMRI_REST1_RL	672756.' ':Day 1	18:35:28	rfMRI_REST1_LR	672756.' ':Day 2	12:32:54	rfMRI_REST2_RL	672756.' ':Day 2	12:48:01	rfMRI_REST2_LR
499566.' ':Day 1	10:54:28	rfMRI_REST1_RL	499566.' ':Day 1	11:09:47	rfMRI_REST1_LR	499566.' ':Day 2	12:06:36	rfMRI_REST2_RL	499566.' ':Day 2	12:21:48	rfMRI_REST2_LR
792564.' ':Day 1	12:55:18	rfMRI_REST1_RL	792564.' ':Day 1	13:10:29	rfMRI_REST1_LR	792564.' ':Day 2	10:48:43	rfMRI_REST2_RL	792564.' ':Day 2	11:03:44	rfMRI_REST2_LR
125525.' ':Day 1	11:18:29	rfMRI_REST1_RL	125525.' ':Day 1	11:33:24	rfMRI_REST1_LR	125525.' ':Day 2	12:49:57	rfMRI_REST2_RL	125525.' ':Day 2	13:04:50	rfMRI_REST2_LR
100408.' ':Day 1	13:39:44	rfMRI_REST1_RL	100408.' ':Day 1	13:55:11	rfMRI_REST1_LR	100408.' ':Day 2	12:20:58	rfMRI_REST2_LR	100408.' ':Day 2	12:35:55	rfMRI_REST2_RL
101107.' ':Day 1	16:56:38	rfMRI_REST1_RL	101107.' ':Day 1	17:11:46	rfMRI_REST1_LR	101107.' ':Day 2	16:05:23	rfMRI_REST2_LR	101107.' ':Day 2	16:20:35	rfMRI_REST2_RL
101309.' ':Day 1	10:27:56	rfMRI_REST1_RL	101309.' ':Day 1	10:43:10	rfMRI_REST1_LR	101309.' ':Day 2	09:47:36	rfMRI_REST2_LR	101309.' ':Day 2	10:02:56	rfMRI_REST2_RL
101915.' ':Day 1	13:43:08	rfMRI_REST1_RL	101915.' ':Day 1	13:58:08	rfMRI_REST1_LR	101915.' ':Day 2	11:32:39	rfMRI_REST2_LR	101915.' ':Day 2	11:48:06	rfMRI_REST2_RL
103111.' ':Day 1	14:34:20	rfMRI_REST1_RL	103111.' ':Day 1	14:49:21	rfMRI_REST1_LR	103111.' ':Day 2	15:31:11	rfMRI_REST2_LR	103111.' ':Day 2	15:46:06	rfMRI_REST2_RL
103414.' ':Day 1	20:23:35	rfMRI_REST1_RL	103414.' ':Day 1	20:38:35	rfMRI_REST1_LR	103414.' ':Day 3	11:59:50	rfMRI_REST2_LR	103414.' ':Day 3	12:15:25	rfMRI_REST2_RL
103818.' ':Day 1	16:39:22	rfMRI_REST1_RL	103818.' ':Day 1	16:54:18	rfMRI_REST1_LR	103818.' ':Day 2	12:38:40	rfMRI_REST2_LR	103818.' ':Day 2	12:53:53	rfMRI_REST2_RL
105014.' ':Day 1	15:47:19	rfMRI_REST1_RL	105014.' ':Day 1	16:03:22	rfMRI_REST1_LR	105014.' ':Day 2	16:25:54	rfMRI_REST2_LR	105014.' ':Day 2	16:41:07	rfMRI_REST2_RL
105115.' ':Day 1	11:15:50	rfMRI_REST1_RL	105115.' ':Day 1	11:30:58	rfMRI_REST1_LR	105115.' ':Day 2	11:11:58	rfMRI_REST2_LR	105115.' ':Day 2	11:26:58	rfMRI_REST2_RL
106016.' ':Day 1	12:07:17	rfMRI_REST1_RL	106016.' ':Day 1	12:22:35	rfMRI_REST1_LR	106016.' ':Day 2	13:26:17	rfMRI_REST2_LR	106016.' ':Day 2	13:41:19	rfMRI_REST2_RL
108828.' ':Day 1	19:10:58	rfMRI_REST1_RL	108828.' ':Day 1	19:26:12	rfMRI_REST1_LR	108828.' ':Day 2	19:33:17	rfMRI_REST2_LR	108828.' ':Day 2	19:48:54	rfMRI_REST2_RL
110411.' ':Day 1	17:45:43	rfMRI_REST1_RL	110411.' ':Day 1	18:00:38	rfMRI_REST1_LR	110411.' ':Day 2	12:09:46	rfMRI_REST2_LR	110411.' ':Day 2	12:24:40	rfMRI_REST2_RL
111716.' ':Day 1	20:35:15	rfMRI_REST1_RL	111716.' ':Day 1	20:50:23	rfMRI_REST1_LR	111716.' ':Day 2	19:53:04	rfMRI_REST2_LR	111716.' ':Day 2	20:08:00	rfMRI_REST2_RL
113619.' ':Day 1	15:25:06	rfMRI_REST1_RL	113619.' ':Day 1	15:40:06	rfMRI_REST1_LR	113619.' ':Day 2	11:02:11	rfMRI_REST2_LR	113619.' ':Day 2	11:17:05	rfMRI_REST2_RL
113922.' ':Day 1	12:02:21	rfMRI_REST1_RL	113922.' ':Day 1	12:17:29	rfMRI_REST1_LR	113922.' ':Day 2	10:56:20	rfMRI_REST2_LR	113922.' ':Day 2	11:11:31	rfMRI_REST2_RL
114419.' ':Day 1	11:47:44	rfMRI_REST1_RL	114419.' ':Day 1	12:03:12	rfMRI_REST1_LR	114419.' ':Day 2	19:06:30	rfMRI_REST2_LR	114419.' ':Day 2	19:21:27	rfMRI_REST2_RL
115320.' ':Day 1	14:37:24	rfMRI_REST1_RL	115320.' ':Day 1	14:52:32	rfMRI_REST1_LR	115320.' ':Day 2	12:39:38	rfMRI_REST2_LR	115320.' ':Day 2	12:54:43	rfMRI_REST2_RL
116524.' ':Day 1	16:04:16	rfMRI_REST1_RL	116524.' ':Day 1	16:19:45	rfMRI_REST1_LR	116524.' ':Day 2	13:12:07	rfMRI_REST2_LR	116524.' ':Day 2	13:27:04	rfMRI_REST2_RL
117122.' ':Day 2	19:49:45	rfMRI_REST1_RL	117122.' ':Day 2	20:04:47	rfMRI_REST1_LR	117122.' ':Day 3	17:46:32	rfMRI_REST2_LR	117122.' ':Day 3	18:01:25	rfMRI_REST2_RL
118528.' ':Day 1	10:58:28	rfMRI_REST1_RL	118528.' ':Day 1	11:13:41	rfMRI_REST1_LR	118528.' ':Day 2	11:57:36	rfMRI_REST2_LR	118528.' ':Day 2	12:12:52	rfMRI_REST2_RL
118730.' ':Day 1	18:28:50	rfMRI_REST1_RL	118730.' ':Day 1	18:43:51	rfMRI_REST1_LR	118730.' ':Day 2	19:03:41	rfMRI_REST2_LR	118730.' ':Day 2	19:18:37	rfMRI_REST2_RL
118932.' ':Day 1	15:32:42	rfMRI_REST1_RL	118932.' ':Day 1	15:47:58	rfMRI_REST1_LR	118932.' ':Day 2	16:19:20	rfMRI_REST2_LR	118932.' ':Day 2	16:34:18	rfMRI_REST2_RL
120111.' ':Day 1	12:17:40	rfMRI_REST1_RL	120111.' ':Day 1	12:32:35	rfMRI_REST1_LR	120111.' ':Day 2	19:21:47	rfMRI_REST2_LR	120111.' ':Day 2	19:36:58	rfMRI_REST2_RL
122317.' ':Day 1	11:21:36	rfMRI_REST1_RL	122317.' ':Day 1	11:36:52	rfMRI_REST1_LR	122317.' ':Day 2	12:44:21	rfMRI_REST2_LR	122317.' ':Day 2	12:59:42	rfMRI_REST2_RL
122620.' ':Day 1	14:18:18	rfMRI_REST1_RL	122620.' ':Day 1	14:33:14	rfMRI_REST1_LR	122620.' ':Day 2	11:01:51	rfMRI_REST2_LR	122620.' ':Day 2	11:16:49	rfMRI_REST2_RL
123117.' ':Day 1	18:37:12	rfMRI_REST1_RL	123117.' ':Day 1	18:52:06	rfMRI_REST1_LR	123117.' ':Day 2	15:48:28	rfMRI_REST2_LR	123117.' ':Day 2	16:03:21	rfMRI_REST2_RL
123925.' ':Day 1	10:18:51	rfMRI_REST1_RL	123925.' ':Day 1	10:34:29	rfMRI_REST1_LR	123925.' ':Day 2	10:38:31	rfMRI_REST2_LR	123925.' ':Day 2	10:53:37	rfMRI_REST2_RL
124422.' ':Day 1	11:10:22	rfMRI_REST1_RL	124422.' ':Day 1	11:25:25	rfMRI_REST1_LR	124422.' ':Day 2	11:21:57	rfMRI_REST2_LR	124422.' ':Day 2	11:36:56	rfMRI_REST2_RL
126325.' ':Day 1	14:16:26	rfMRI_REST1_RL	126325.' ':Day 1	14:31:25	rfMRI_REST1_LR	126325.' ':Day 2	16:07:21	rfMRI_REST2_LR	126325.' ':Day 2	16:22:14	rfMRI_REST2_RL
127630.' ':Day 1	12:29:22	rfMRI_REST1_RL	127630.' ':Day 1	12:44:42	rfMRI_REST1_LR	127630.' ':Day 2	11:55:52	rfMRI_REST2_LR	127630.' ':Day 2	12:10:57	rfMRI_REST2_RL
127933.' ':Day 1	09:27:56	rfMRI_REST1_RL	127933.' ':Day 1	09:42:51	rfMRI_REST1_LR	127933.' ':Day 2	09:37:59	rfMRI_REST2_LR	127933.' ':Day 2	09:52:54	rfMRI_REST2_RL
128127.' ':Day 1	12:17:44	rfMRI_REST1_RL	128127.' ':Day 1	12:33:04	rfMRI_REST1_LR	128127.' ':Day 2	12:14:24	rfMRI_REST2_LR	128127.' ':Day 2	12:30:03	rfMRI_REST2_RL
128632.' ':Day 1	10:53:12	rfMRI_REST1_RL	128632.' ':Day 1	11:09:15	rfMRI_REST1_LR	128632.' ':Day 2	10:51:33	rfMRI_REST2_LR	128632.' ':Day 2	11:06:46	rfMRI_REST2_RL
129028.' ':Day 1	12:43:26	rfMRI_REST1_RL	129028.' ':Day 1	12:58:31	rfMRI_REST1_LR	129028.' ':Day 2	11:51:42	rfMRI_REST2_LR	129028.' ':Day 2	12:06:51	rfMRI_REST2_RL
130013.' ':Day 2	13:51:00	rfMRI_REST1_RL	130013.' ':Day 2	14:06:03	rfMRI_REST1_LR	130013.' ':Day 3	10:05:29	rfMRI_REST2_LR	130013.' ':Day 3	10:20:26	rfMRI_REST2_RL
130316.' ':Day 1	13:21:33	rfMRI_REST1_RL	130316.' ':Day 1	13:36:27	rfMRI_REST1_LR	130316.' ':Day 2	15:21:39	rfMRI_REST2_LR	130316.' ':Day 2	15:36:35	rfMRI_REST2_RL
131217.' ':Day 1	14:49:53	rfMRI_REST1_RL	131217.' ':Day 1	15:05:05	rfMRI_REST1_LR	131217.' ':Day 2	10:04:18	rfMRI_REST2_LR	131217.' ':Day 2	10:19:18	rfMRI_REST2_RL
131722.' ':Day 2	08:41:53	rfMRI_REST1_RL	131722.' ':Day 2	08:57:04	rfMRI_REST1_LR	131722.' ':Day 3	12:28:11	rfMRI_REST2_LR	131722.' ':Day 3	12:43:41	rfMRI_REST2_RL
133019.' ':Day 1	12:16:53	rfMRI_REST1_RL	133019.' ':Day 1	12:31:57	rfMRI_REST1_LR	133019.' ':Day 2	10:59:20	rfMRI_REST2_LR	133019.' ':Day 2	11:14:42	rfMRI_REST2_RL
133928.' ':Day 1	11:17:51	rfMRI_REST1_RL	133928.' ':Day 1	11:33:09	rfMRI_REST1_LR	133928.' ':Day 2	11:49:29	rfMRI_REST2_LR	133928.' ':Day 2	12:04:44	rfMRI_REST2_RL
135225.' ':Day 1	11:40:36	rfMRI_REST1_RL	135225.' ':Day 1	11:55:46	rfMRI_REST1_LR	135225.' ':Day 2	10:59:46	rfMRI_REST2_LR	135225.' ':Day 2	11:15:55	rfMRI_REST2_RL
135932.' ':Day 1	15:40:11	rfMRI_REST1_RL	135932.' ':Day 1	15:55:29	rfMRI_REST1_LR	135932.' ':Day 2	11:19:05	rfMRI_REST2_LR	135932.' ':Day 2	11:34:13	rfMRI_REST2_RL
136833.' ':Day 1	15:33:04	rfMRI_REST1_RL	136833.' ':Day 1	15:48:02	rfMRI_REST1_LR	136833.' ':Day 2	12:29:02	rfMRI_REST2_LR	136833.' ':Day 2	12:44:15	rfMRI_REST2_RL
138534.' ':Day 1	12:49:36	rfMRI_REST1_RL	138534.' ':Day 1	13:04:59	rfMRI_REST1_LR	138534.' ':Day 2	11:33:40	rfMRI_REST2_LR	138534.' ':Day 2	11:48:34	rfMRI_REST2_RL
139637.' ':Day 1	11:19:04	rfMRI_REST1_RL	139637.' ':Day 1	11:34:33	rfMRI_REST1_LR	139637.' ':Day 2	11:18:28	rfMRI_REST2_LR	139637.' ':Day 2	11:33:58	rfMRI_REST2_RL
140925.' ':Day 1	12:33:47	rfMRI_REST1_RL	140925.' ':Day 1	12:48:50	rfMRI_REST1_LR	140925.' ':Day 2	09:58:15	rfMRI_REST2_LR	140925.' ':Day 2	10:13:14	rfMRI_REST2_RL
144832.' ':Day 1	14:33:29	rfMRI_REST1_RL	144832.' ':Day 1	14:48:51	rfMRI_REST1_LR	144832.' ':Day 2	14:09:08	rfMRI_REST2_LR	144832.' ':Day 2	14:25:14	rfMRI_REST2_RL
146432.' ':Day 1	13:35:59	rfMRI_REST1_RL	146432.' ':Day 1	13:51:00	rfMRI_REST1_LR	146432.' ':Day 2	11:28:40	rfMRI_REST2_LR	146432.' ':Day 2	11:44:02	rfMRI_REST2_RL
147737.' ':Day 1	12:16:17	rfMRI_REST1_RL	147737.' ':Day 1	12:31:25	rfMRI_REST1_LR	147737.' ':Day 2	12:12:53	rfMRI_REST2_LR	147737.' ':Day 2	12:27:50	rfMRI_REST2_RL
148335.' ':Day 1	16:38:15	rfMRI_REST1_RL	148335.' ':Day 1	16:53:18	rfMRI_REST1_LR	148335.' ':Day 2	13:57:56	rfMRI_REST2_LR	148335.' ':Day 2	14:12:54	rfMRI_REST2_RL
148840.' ':Day 1	19:16:09	rfMRI_REST1_RL	148840.' ':Day 1	19:31:12	rfMRI_REST1_LR	148840.' ':Day 2	19:08:36	rfMRI_REST2_LR	148840.' ':Day 2	19:23:31	rfMRI_REST2_RL
149337.' ':Day 1	17:49:53	rfMRI_REST1_RL	149337.' ':Day 1	18:04:55	rfMRI_REST1_LR	149337.' ':Day 2	17:04:58	rfMRI_REST2_LR	149337.' ':Day 2	17:20:14	rfMRI_REST2_RL
149539.' ':Day 1	16:37:38	rfMRI_REST1_RL	149539.' ':Day 1	16:52:46	rfMRI_REST1_LR	149539.' ':Day 2	15:08:14	rfMRI_REST2_LR	149539.' ':Day 2	15:23:24	rfMRI_REST2_RL
149741.' ':Day 1	16:29:01	rfMRI_REST1_RL	149741.' ':Day 1	16:44:15	rfMRI_REST1_LR	149741.' ':Day 2	16:11:01	rfMRI_REST2_LR	149741.' ':Day 2	16:26:00	rfMRI_REST2_RL
151223.' ':Day 1	13:54:03	rfMRI_REST1_RL	151223.' ':Day 1	14:09:29	rfMRI_REST1_LR	151223.' ':Day 2	11:17:39	rfMRI_REST2_LR	151223.' ':Day 2	11:32:47	rfMRI_REST2_RL
151526.' ':Day 1	10:07:31	rfMRI_REST1_RL	151526.' ':Day 1	10:22:50	rfMRI_REST1_LR	151526.' ':Day 2	10:28:29	rfMRI_REST2_LR	151526.' ':Day 2	10:43:48	rfMRI_REST2_RL
151627.' ':Day 1	14:01:20	rfMRI_REST1_RL	151627.' ':Day 1	14:16:32	rfMRI_REST1_LR	151627.' ':Day 2	12:09:14	rfMRI_REST2_LR	151627.' ':Day 2	12:24:24	rfMRI_REST2_RL
153025.' ':Day 1	17:03:35	rfMRI_REST1_RL	153025.' ':Day 1	17:18:44	rfMRI_REST1_LR	153025.' ':Day 2	16:53:43	rfMRI_REST2_LR	153025.' ':Day 2	17:08:59	rfMRI_REST2_RL
154734.' ':Day 1	10:31:22	rfMRI_REST1_RL	154734.' ':Day 1	10:46:21	rfMRI_REST1_LR	154734.' ':Day 2	12:09:52	rfMRI_REST2_LR	154734.' ':Day 2	12:24:48	rfMRI_REST2_RL
156637.' ':Day 1	11:22:19	rfMRI_REST1_RL	156637.' ':Day 1	11:37:56	rfMRI_REST1_LR	156637.' ':Day 2	08:37:11	rfMRI_REST2_LR	156637.' ':Day 2	08:52:21	rfMRI_REST2_RL
159340.' ':Day 1	18:50:51	rfMRI_REST1_RL	159340.' ':Day 1	19:05:59	rfMRI_REST1_LR	159340.' ':Day 2	11:25:20	rfMRI_REST2_LR	159340.' ':Day 2	11:40:51	rfMRI_REST2_RL
160123.' ':Day 1	10:58:00	rfMRI_REST1_RL	160123.' ':Day 1	11:13:05	rfMRI_REST1_LR	160123.' ':Day 2	11:11:53	rfMRI_REST2_LR	160123.' ':Day 2	11:26:57	rfMRI_REST2_RL
161731.' ':Day 1	18:22:10	rfMRI_REST1_RL	161731.' ':Day 1	18:37:23	rfMRI_REST1_LR	161731.' ':Day 2	12:44:42	rfMRI_REST2_LR	161731.' ':Day 2	12:59:48	rfMRI_REST2_RL
162733.' ':Day 1	13:08:35	rfMRI_REST1_RL	162733.' ':Day 1	13:23:44	rfMRI_REST1_LR	162733.' ':Day 2	13:02:08	rfMRI_REST2_LR	162733.' ':Day 2	13:17:04	rfMRI_REST2_RL
163129.' ':Day 1	18:40:27	rfMRI_REST1_RL	163129.' ':Day 1	18:55:48	rfMRI_REST1_LR	163129.' ':Day 2	09:41:47	rfMRI_REST2_LR	163129.' ':Day 2	09:56:50	rfMRI_REST2_RL
176542.' ':Day 1	13:33:06	rfMRI_REST1_RL	176542.' ':Day 1	13:48:15	rfMRI_REST1_LR	176542.' ':Day 2	12:07:11	rfMRI_REST2_LR	176542.' ':Day 2	12:22:16	rfMRI_REST2_RL
178950.' ':Day 1	19:22:04	rfMRI_REST1_RL	178950.' ':Day 1	19:37:16	rfMRI_REST1_LR	178950.' ':Day 2	17:33:10	rfMRI_REST2_LR	178950.' ':Day 2	17:48:12	rfMRI_REST2_RL
188347.' ':Day 1	16:04:32	rfMRI_REST1_RL	188347.' ':Day 1	16:19:30	rfMRI_REST1_LR	188347.' ':Day 2	15:24:11	rfMRI_REST2_LR	188347.' ':Day 2	15:39:06	rfMRI_REST2_RL
189450.' ':Day 1	11:57:38	rfMRI_REST1_RL	189450.' ':Day 1	12:12:45	rfMRI_REST1_LR	189450.' ':Day 2	16:25:35	rfMRI_REST2_LR	189450.' ':Day 2	16:40:35	rfMRI_REST2_RL
190031.' ':Day 1	14:24:01	rfMRI_REST1_RL	190031.' ':Day 1	14:39:25	rfMRI_REST1_LR	190031.' ':Day 2	11:09:06	rfMRI_REST2_LR	190031.' ':Day 2	11:24:24	rfMRI_REST2_RL
192540.' ':Day 1	12:06:24	rfMRI_REST1_RL	192540.' ':Day 1	12:21:32	rfMRI_REST1_LR	192540.' ':Day 2	11:19:25	rfMRI_REST2_LR	192540.' ':Day 2	11:34:25	rfMRI_REST2_RL
196750.' ':Day 1	13:18:12	rfMRI_REST1_RL	196750.' ':Day 1	13:33:21	rfMRI_REST1_LR	196750.' ':Day 2	12:54:38	rfMRI_REST2_LR	196750.' ':Day 2	13:09:39	rfMRI_REST2_RL
198451.' ':Day 1	11:21:30	rfMRI_REST1_RL	198451.' ':Day 1	11:36:40	rfMRI_REST1_LR	198451.' ':Day 2	11:04:28	rfMRI_REST2_LR	198451.' ':Day 2	11:19:31	rfMRI_REST2_RL
199655.' ':Day 1	11:17:36	rfMRI_REST1_RL	199655.' ':Day 1	11:33:00	rfMRI_REST1_LR	199655.' ':Day 2	17:52:11	rfMRI_REST2_LR	199655.' ':Day 2	18:07:18	rfMRI_REST2_RL
201111.' ':Day 1	14:40:34	rfMRI_REST1_RL	201111.' ':Day 1	14:55:39	rfMRI_REST1_LR	201111.' ':Day 2	15:34:32	rfMRI_REST2_LR	201111.' ':Day 2	15:49:34	rfMRI_REST2_RL
208226.' ':Day 1	10:46:34	rfMRI_REST1_RL	208226.' ':Day 1	11:01:44	rfMRI_REST1_LR	208226.' ':Day 2	10:57:46	rfMRI_REST2_LR	208226.' ':Day 2	11:12:49	rfMRI_REST2_RL
211417.' ':Day 1	19:34:52	rfMRI_REST1_RL	211417.' ':Day 1	19:49:47	rfMRI_REST1_LR	211417.' ':Day 2	19:05:16	rfMRI_REST2_LR	211417.' ':Day 2	19:20:11	rfMRI_REST2_RL
211720.' ':Day 1	18:03:44	rfMRI_REST1_RL	211720.' ':Day 1	18:18:58	rfMRI_REST1_LR	211720.' ':Day 2	16:04:56	rfMRI_REST2_LR	211720.' ':Day 2	16:20:06	rfMRI_REST2_RL
212318.' ':Day 1	17:01:21	rfMRI_REST1_RL	212318.' ':Day 1	17:16:27	rfMRI_REST1_LR	212318.' ':Day 3	09:02:45	rfMRI_REST2_LR	212318.' ':Day 3	09:17:43	rfMRI_REST2_RL
214423.' ':Day 1	11:52:19	rfMRI_REST1_RL	214423.' ':Day 1	12:07:36	rfMRI_REST1_LR	214423.' ':Day 2	11:02:32	rfMRI_REST2_LR	214423.' ':Day 2	11:17:36	rfMRI_REST2_RL
221319.' ':Day 1	13:55:37	rfMRI_REST1_RL	221319.' ':Day 1	14:11:22	rfMRI_REST1_LR	221319.' ':Day 2	16:25:54	rfMRI_REST2_LR	221319.' ':Day 2	16:41:04	rfMRI_REST2_RL
239944.' ':Day 1	12:50:43	rfMRI_REST1_RL	239944.' ':Day 1	13:05:48	rfMRI_REST1_LR	239944.' ':Day 2	12:05:35	rfMRI_REST2_LR	239944.' ':Day 2	12:20:41	rfMRI_REST2_RL
245333.' ':Day 1	12:55:04	rfMRI_REST1_RL	245333.' ':Day 1	13:10:31	rfMRI_REST1_LR	245333.' ':Day 2	12:23:49	rfMRI_REST2_LR	245333.' ':Day 2	12:38:54	rfMRI_REST2_RL
280739.' ':Day 1	20:49:14	rfMRI_REST1_RL	280739.' ':Day 1	21:04:40	rfMRI_REST1_LR	280739.' ':Day 2	10:01:58	rfMRI_REST2_LR	280739.' ':Day 2	10:16:52	rfMRI_REST2_RL
298051.' ':Day 1	10:40:41	rfMRI_REST1_RL	298051.' ':Day 1	10:55:50	rfMRI_REST1_LR	298051.' ':Day 2	10:31:36	rfMRI_REST2_LR	298051.' ':Day 2	10:46:35	rfMRI_REST2_RL
366446.' ':Day 1	12:41:06	rfMRI_REST1_RL	366446.' ':Day 1	12:56:17	rfMRI_REST1_LR	366446.' ':Day 2	13:40:23	rfMRI_REST2_LR	366446.' ':Day 2	13:55:39	rfMRI_REST2_RL
397760.' ':Day 1	16:30:06	rfMRI_REST1_RL	397760.' ':Day 1	16:45:02	rfMRI_REST1_LR	397760.' ':Day 2	15:37:18	rfMRI_REST2_LR	397760.' ':Day 2	15:52:14	rfMRI_REST2_RL
414229.' ':Day 1	11:10:13	rfMRI_REST1_RL	414229.' ':Day 1	11:25:16	rfMRI_REST1_LR	414229.' ':Day 2	11:06:43	rfMRI_REST2_LR	414229.' ':Day 2	11:21:48	rfMRI_REST2_RL
654754.' ':Day 1	12:26:39	rfMRI_REST1_RL	654754.' ':Day 1	12:41:57	rfMRI_REST1_LR	654754.' ':Day 2	11:00:46	rfMRI_REST2_LR	654754.' ':Day 2	11:15:55	rfMRI_REST2_RL
751348.' ':Day 1	19:11:05	rfMRI_REST1_RL	751348.' ':Day 1	19:26:28	rfMRI_REST1_LR	751348.' ':Day 2	11:26:48	rfMRI_REST2_LR	751348.' ':Day 2	11:41:57	rfMRI_REST2_RL
756055.' ':Day 1	11:48:57	rfMRI_REST1_RL	756055.' ':Day 1	12:07:10	rfMRI_REST1_LR	756055.' ':Day 2	11:00:48	rfMRI_REST2_LR	756055.' ':Day 2	11:15:50	rfMRI_REST2_RL
856766.' ':Day 1	09:46:18	rfMRI_REST1_RL	856766.' ':Day 1	10:01:30	rfMRI_REST1_LR	856766.' ':Day 2	15:43:08	rfMRI_REST2_LR	856766.' ':Day 2	15:58:08	rfMRI_REST2_RL
857263.' ':Day 1	16:17:25	rfMRI_REST1_RL	857263.' ':Day 1	16:32:25	rfMRI_REST1_LR	857263.' ':Day 2	14:53:54	rfMRI_REST2_LR	857263.' ':Day 2	15:08:58	rfMRI_REST2_RL
899885.' ':Day 1	15:03:14	rfMRI_REST1_RL	899885.' ':Day 1	15:18:14	rfMRI_REST1_LR	899885.' ':Day 2	17:06:53	rfMRI_REST2_LR	899885.' ':Day 2	17:21:57	rfMRI_REST2_RL

Supplementary Table ST1 List of HCP U100 subject IDs and respective scan times that were used in this study

Year	Study focus	Measure	Abbr.	Reference
2010	Temporal connectivity of the posterior cingulate cortex within the default mode network, and with task-positive networks	Wavelet transform coherence	WTC	(Chang and Glover, 2010)
2011	Spectral and phase characteristics of resting state networks	Mean phase	PS	(Niazy et al., 2011)
2012	Time-varying FC, and time-varying inter-subject reliability	Mean phase-locking	PLV	(Glerean et al., 2012)
2015	Temporal patterns within and between intrinsic connectivity networks for eyes-open and eyes-closed conditions	Mean phase	PS	(Wang et al., 2015)
2015	Spatiotemporal dynamics of phase interactions among resting-state fMRI signals	Mean phase-locking	PS	(Ponce-Alvarez et al., 2015)
2017	Time dependency of envelope correlations between fMRI and MEG signals	Cosine relative phase	PL	(Deco et al., 2017)
2017	Cognitive performance in healthy older adults	Cosine relative phase	PL	(Cabral et al., 2017)
2018	The relationship between instantaneous phase synchrony and correlation-based sliding	Phase coherence $(1 - \lvert\sin(\Delta\boldsymbol{\theta}(t))\rvert)$	PC	(Pederson, 2018)
2019	Patients remitted from major depressive disorder	Cosine relative phase	PL	(Figueroa et al., 2019)
2019	Temporal dynamics of phase synchronization	Mean phase	PS	(Nobukawa et al., 2019)
2020	Depressive symptoms in a nonclinical sample	Cosine relative phase	PL	(Alonso Martínez et al., 2020)
2020	A detailed characterization of the dynamical features of functional networks	Cosine relative phase	PL	(Vohryzek et al., 2020)
2021	Temporal summation of pain in fibromyalgia	Mean phase	PS	(Cheng et al., 2021)
2021	Evaluation of phase synchronization methods in fMRI	Cosine relative phase	PL	(Honari et al., 2021)
2022	Characterization and prediction of individual traits were investigated	Phase coherence $(1 - \lvert\sin(\Delta\boldsymbol{\theta}(t))\rvert)$	PC	(Cai et al., 2022)

Supplementary Table ST2 Overview of dFC studies since 2010 where measures of phase synchronization were employed to compute dynamic functional connectivity.

Measure	Relevance (T-theoretical, E-empirical)	Reference
**Phase-Locking**		
Fractional occurrence	Differences associated with major depression (E)	(Alonso Martínez et al., 2020)
Duration	Differences associated with Alzheimer's disease (E)	(Sendi et al., 2021)
Typical FC reconfiguration speed	Slows down in older healthy adults (E)	(Battaglia et al., 2020)
Fractal scaling coefficient	Reduces with increasing age (E)	(Battaglia et al., 2020)
**Phase Synchronization**		
Synchronization	Healthy cortex operates with moderate synchrony (E,T)	(Yang et al., 2012)
Metastability	Increases with psilocybin (E)	(Lord et al., 2019)
Cluster synchronization (Chimera Index)	Requires a certain degree of metastability (T)	(Cabral et al., 2014)
Diversity of cluster synchronization (Coalition entropy)	Occurs at transition between global synchronization and disorder (T)	(Wildie and Shanahan, 2012)
Phase coherence across synchronized communities	Requires connectivity between communities (T)<br>Required for CTC-like channels of communication (T)	(Wildie and Shanahan, 2012)<br>(Deco and Kringelbach, 2016)
Integrated Information	Loss of consciousness reduced integrated information (E)	(Luppi et al., 2020)

Supplementary Table ST3 Examples of application of dFC metrics in empirical and theoretical studies

META			SYNC			CHI		
group	statistic	p.value	group	statistic	p.value	group	statistic	p.value
RUN1	0.979	0.116	RUN1	0.975	0.056	RUN1	0.992	0.834
RUN2	0.996	0.988	RUN2	0.990	0.674	RUN2	0.984	0.262
RUN3	0.981	0.162	RUN3	0.988	0.497	RUN3	0.994	0.955
RUN4	0.978	0.092	RUN4	0.992	0.826	RUN4	0.990	0.648
OCC			DUR			CENTROPY		
group	statistic	p.value	group	statistic	p.value	group	statistic	p.value
RUN1	0.961	0.005	RUN1	0.903	0.000	RUN1	0.971	0.029
RUN2	0.979	0.121	RUN2	0.885	0.000	RUN2	0.988	0.537
RUN3	0.985	0.334	RUN3	0.923	0.000	RUN3	0.989	0.627
RUN4	0.974	0.047	RUN4	0.940	0.000	RUN4	0.989	0.594
PHI			PCC			SPEED		
group	statistic	p.value	group	statistic	p.value	group	statistic	p.value
RUN1	0.900	0.000	RUN1	0.852	0.000	RUN1	0.988	0.511
RUN2	0.955	0.002	RUN2	0.942	0.000	RUN2	0.987	0.438
RUN3	0.944	0.000	RUN3	0.947	0.001	RUN3	0.979	0.115
RUN4	0.953	0.001	RUN4	0.945	0.000	RUN4	0.986	0.387

Supplementary Table ST4 Shapiro-Wilk test statistics for global metrics in 4 scans

B Supplementary methods and metrics

Dynamical systems metrics

Fractional occurrence

For each subject we assigned every timepoint to its closest centroid V_c, providing a subject specific mode time-course. This time-course is the basis for the calculation of two statistical metrics as follow:

Fractional occurrence of mode ψ was calculated as

$$\Pi_{\psi}^{(S)} = \frac{1}{T}\sum_{t=1}^{T} \mathcal{X}\left[x^{*}(t) \in R^{\psi}\right] \tag{S.1}$$

where $\mathcal{X}$ is a binary event indicator, T is the number of time points in each scan S, x^{*} represents the trajectory, or cluster time-series, and R^{ψ} is the defined cluster under consideration.

Duration

Duration was calculated as the mean of all consecutive periods spent in a particular mode

$$D_{\psi} = \frac{1}{p_{\psi}}\sum\nolimits_{1}^{p_{\psi}} C_{p_{\psi}} \tag{S.2}$$

where D_{ψ} is the duration of mode ψ, p_{ψ} is the number of consecutive periods, and $C_{p_{\psi}}$ is the duration of each consecutive period in each scan S.

Stochastic processes - dFC speed analysis

An unanswered question regarding dFC is whether spatiotemporal patterns change in a discrete or continuous manner over time. K-means clustering yields a

distinct mode for each timepoint, but this mode is just the dominant mode, defined to be the mode with the shortest distance to the cluster centroid, and a number of other modes may also contribute to the resulting spatiotemporal pattern at that timepoint. An alternative perspective is to view dFC as a smooth reconfiguration of phase-related connectivity, and to collapse these relations to a point in the space of possible relations. We can then view the evolution of this point as a stochastic exploration of a high-dimensional space. This approach is a direct adaptation of (Battaglia et al., 2020) for phase-locking. The stochastic exploration, or random walk, is quantitatively characterized by its typical reconfiguration speed and path shape (Battaglia et al., 2020).

Following (Battaglia et al., 2020) and (Hansen et al., 2015) we adopted a notion of similarity between $iPL(t)$ matrices based on the Pearson correlation between their upper triangular elements.

$$dPL(t_1, t_2) = corr\big[(UpperTri\big(iPL(t_1)\big), UpperTri(iPL(t_2))\big] \tag{S.3}$$

The instantaneous global dPL reconfiguration speed at time t is then

$$Speed_{dPL}(t) = 1 - dPL(t_1, t_2) \tag{S.4}$$

The typical reconfiguration speed is taken as the median of the instantaneous velocity

$$Speed_{typ} = median(Speed_{dPL})_G \tag{S.5}$$

We used Pearson correlation rather than cosine similarity (Cabral et al., 2017) to retain close alignment with (Battaglia et al., 2020) and (Lombardo et al., 2020).

The same calculations were performed for each spatiotemporal mode ψ returned with K-means clustering analogous with (Lombardo et al., 2020).

To visualize the random walk in 3 dimensions, the sequence of time-dependent phase-locking matrices, or *iPL stream*, served as input features into a t-Stochastic Neighborhood Embedding (t-SNE) algorithm (algorithm = exact, distance = Cosine, Perplexity = 50, LearnRate =2000, exaggeration = 4) in MATLAB (MathWorks R2020b) following (Battaglia et al., 2020; Maaten and Hinton, 2008).

Detrended fluctuation analysis

Detrended Fluctuation Analysis (DFA) is a method to detect intrinsic statistical self-affinity embedded in a time series. Self-affinity is a property of fractal timeseries where the similarity is anisotropic. Statistical fractals refer to self-similarity expressed in terms of statistical properties (Eke et al., 2002). DFA infers a self-affinity coefficient through the comparison of detrended mean square fluctuations of the integrated signal over a range of observation scales in a log-log plot. If a genuine power-law exists over a continuous range of scales, then the log-log plot will have an extended linear section, meaning that fluctuations are similar across different temporal scales. So statistically, we have the same fluctuations if we scale the intensity of the signal with the computed DFA coefficient (Eke et al., 2002).

DFA was employed to characterize the shape of the random walk path. Stochastic processes are descriptions of random phenomena evolving over time that are governed by certain laws of probability. Two specific stochastic processes are of relevance for us: the Gaussian process, which represents random noise; and the Wiener process or Brownian motion, which represents a temporally homogenous additive process (Kakihara, 2003). According to the Signal Summation Conversion (SSC) (Eke et al., 2002), the signal (reconfiguration speed in our case) is seen as the realization of either fractional Gaussian noise (fGn) or fractional Brownian motion

(fBn). The fBn is non-stationary with independent stationary increments and underlies the computation of DFA. According to SSC, a fGn signal is converted to an fBn through summation before performance of DFA (Eke et al., 2002). We used the fluctuation analysis toolbox of (Ton and Daffertshofer, 2016) available at https://github.com/marlow17/FluctuationAnalysis to perform the DFA.

The time series of instantaneous dPL speed increments was converted into a fBn signal by computing its cumulative sum (Eke et al., 2000):

$$D\,(t_L) = \sum_{l=1}^{L} Speed_{dPL(t_l)} \tag{S.6}$$

Let K denote the number of time series samples split into Q non-overlapping segments $q = 1 \dots Q$ of length k, with $Q = K/k$. For each segment q, the fluctuation strength F_q was computed as the squared difference between $D\,(t)$ and its trend $D^{(trend)}(t)$ (this is the regression line of $D(t)$ over the interval $t = 1 \dots k$ in the linear case).

$$F_q^2(k) = \frac{1}{k}\sum_{l=0}^{k-l}\left[D\left(t_{(q+l)}\right) - D^{(trend)}(t_{(q+l)})\right]^2 \tag{S.7}$$

This fluctuation strength scales with segment size k in the case of scale-free correlation. On average a linear power law is found in the form of

$$\log F_q\,(k) = \alpha_{DFA} log\; k + C \tag{S.8}$$

The scaling parameter α_{DFA} resembles the Hurst exponent (Metzler et al., 2014) and may be interpreted as follows:

$0 < \alpha_{DFA} < 0.5: Speed_{dPL}(t)$ displays anti-persistent fluctuations

$\alpha_{DFA} = 0.5: Speed_{dPL}(t)$	displays uncorrelated Gaussian fluctuations
$0.5 < \alpha_{DFA} < 1: Speed_{dPL}(t)$	displays persistent fluctuations (approaching pink noise when α_{DFA} is close to 1)
$1 \leq \alpha_{DFA}: Speed_{dPL}(t)$	is non-stationary

Power-law scaling test

Returning to the subject of linear power-law scaling, it is imperative to verify that scaling is indeed linear. If this is not the case, the DFA coefficient cannot be interpreted as a scaling exponent (Eke et al., 2000). Following (Ton and Daffertshofer, 2016), we tested the hypothesis of power-law scaling using a Bayesian model comparison approach. This allowed us to fit each subject's DFA log-log plot with 11 different models. Only subjects with an extended linear section over the complete timeseries, returned as a best fit with the linear model, were retained for metric analysis. These subjects were considered to have a 'genuine' DFA_{α} exponent.

The DFA analysis toolbox evaluates the density of fluctuations over consecutive segments using a kernel source density estimator, and then estimates the log-likelihood for a certain model to generate fluctuations of a given strength (on a log-scale) as a function of log k. Model selection is then performed through computation of the Bayesian Information Criteria (BIC) for each of the tested models defined as

$$BIC = -2log(\mathcal{L}_{\max}) + plog(Q) \qquad \text{(S.9)}$$

Where p represents the number of parameters of the model under study, Q the number of intervals with different length k, and $\mathcal{L}_{max}$ denotes the maximum value of the likelihood function $\mathcal{L}$, which quantifies the goodness-of-fit. The model yielding

the lowest BIC was selected as the best one, and if it corresponded to model 1 (the linear model), then the returned DFA value was taken as DFA_α.

In our analysis of 4 runs, we could only confirm linear power-law scaling over the complete timeseries for between 60% to 70% of our 99 subjects in AAL90 parcellation, and 50% to 60% in AAL116. Of these subjects, less than 10 exhibited power-law scaling over all 4 runs. Consequently, we did not include DFA_α as a potential reliable metric for dFC analysis, but we did use the average value of 'genuine' DFA_α exponents to assess the nature of the fluctuations. We will however, follow up on these intriguing results in a future study, as non-linear power-law scaling, in many cases due to a drop in linearity towards the end of the timeseries, has been found elsewhere in certain subjects (Botcharova, 2014).

Parameter selection

The toolbox for random walk analysis (Arbabyazd et al., 2020) was developed for dFC computed with temporal correlations and sliding windows. We referred to previous studies using DFA to identify appropriate parameters for use with phase related measures (Botcharova, 2014; Daffertshofer et al., 2018; Eke et al., 2002; Hardstone et al., 2012; Tagliazucchi et al., 2016, 2013; Tetereva et al., 2020; Ton and Daffertshofer, 2016). Consequently, parameters used with the function FluctuationAnalysis() (Ton and Daffertshofer, 2016) were set as follows: minimum segment length 10 (Tagliazucchi et al., 2013), maximum segment length of Tmax /2 where Tmax is the number of TRs in the scan (Tagliazucchi et al., 2013; Ton and Daffertshofer, 2016), and number of partitions within a segment 20 (Ton and Daffertshofer, 2016).

Phase synchrony analysis

Synergetics and Kuramoto

Synergetics is an advanced theory of the phenomena of self-organization where it is proposed that the brain operates close to instabilities otherwise referred to as phase transitions (Haken, 1989). A phase transition occurs when a system undergoes a qualitative change in its macroscopic order. Leveraging the center manifold theorem in dynamical systems theory, the behavior of a system in the vicinity of a phase transition can be approximated by low-dimensional dynamical systems described by a set of order parameter equations. In the Kuramoto model of weakly-coupled interacting limit-cycle oscillators, the collective dynamics of the entire population of oscillators is measured by the macroscopic complex order parameter (Arenas et al., 2008). Here we quantify metastability as the variability in the Kuramoto order parameter (Wildie and Shanahan, 2012) as previous research has demonstrated that this variability is linked to the underlying metastable cluster synchronization in a system of weakly coupled oscillators (Cabral et al., 2011).

Empirical metastability studies to date have used pre-defined resting-state networks (RSN) extracted with ICA (Hellyer et al., 2014), with network masks (Lee et al., 2018), or with functional templates (Lee et al., 2019) to represent communities of oscillators for investigation of network synchrony and metastability. In contrast, we decided to take a purely data driven approach, using the recurrent modes extracted with K-means clustering to represent communities of oscillators. As we decided to retain 5 recurrent modes, we have 5 communities of oscillators ψ_1 - ψ_5. Note that the AAL regions are not constrained to a single community and so the communities reflect time-varying coalitions among regions. The Kuramoto order parameters for any mode ψ provide a quantification of the order among the phases over time. Considering the mean of the phases $\theta(r,t)$ across all areas r in a given community ψ,

$$Z_{\psi}(t) = \langle e^{i\theta(r,t)} \rangle_{,\ r \in \psi} \qquad \text{(S.10)}$$

$Z_{\psi}(t)$ is a complex value where its magnitude, $SYNC_{\psi}= |Z_{\psi}(t)|$, provides a quantification of the degree of synchronization of the community at each time t, whereas its phase, $PHASE_{\psi}(t) = \arg\left(Z_{\psi}(t)\right)$, indicates the main direction of the phases in the community at each time t, which becomes more meaningful as the magnitude of the order parameter increases.

The mean synchrony over time $\langle SYNC_{\psi} \rangle_{T}$ provides a quantification of the average degree of synchronization of the community over the complete scan duration. SYNC_{ψ} takes values between 0 and 1, reflecting phase alignment The standard deviation of the synchrony over time, $\mathrm{META}_{\psi} = \sigma(SYNC_{\psi})_{T}$, provides a measure of metastability of the mode ψ over the complete scan duration.

If we fix time t and estimate the variance of $Z_{C}(t)$ over the set of all communities C, we obtain an instantaneous measure of how chimera-like the system is at time t.

$$CHI_{c}(t) = var\left(e^{i\theta(c,t)}\right)_{c\in C} \tag{S.11}$$

where a measure of the chimera index, an indicator of cluster synchronization, over the complete scan duration is provided by

$$\chi_{G} = \langle CHI \rangle_{G_{T}} \tag{S.12}$$

The instantaneous phase-coherence of highly synchronized communities at time t is given by

$$IPC_{S} = \left|\langle e^{iPHASE_{\psi}(t)} \rangle_{\psi\in S}\right| \tag{S.13}$$

where $\psi \in S$ if $SYNC_{\psi} > \lambda$ at time t , and $|S| > 1$. The synchronization threshold λ was set to 0.8 (Wildie & Shanahan, 2012).

Global META was calculated as $\langle \text{META} \rangle_{\psi}$ and global SYNC was calculated as the mean value for the global mode ψ_1, $\langle \text{SYNC} \rangle_{\psi_1}$ as this mode includes all regions of all communities. The Phase-Coherence Coefficient (PCC) was calculated as the fractional amount of time that IPC_S was non-zero over the complete scan duration.

Information dynamics analysis

Metastability has been described as a subtle blend of segregation and integration among brain regions that show tendencies to diverge and function independently, with tendencies to converge and function collectively (Tognoli and Kelso, 2014). The simultaneous presence of these complementary tendencies implies that information shared between regions will either be differentiated to allow for independent processing or integrated for whole brain behavior. Contingent on the premise that the brain can be comprehensively characterized by its information dynamics (Tononi et al., 1994), the brain's current state contains information about its past and future states (in terms of predictability). A measure of integrated information was first proposed (Balduzzi and Tononi, 2008).

$$\Phi = I\left(X_{t;}\, X_{t+1}\right) - \sum_{i=1}^{n} I(X_t^i;\, X_{t+1}^i) \qquad \text{(S.14)}$$

Where X_t denotes the state of the whole system at time t; X_t^i denotes the ith part of X at time t. Φ or Integrated Information, captures the information flow between the past and future observed in the whole system X, with the flow observed within each of its part, or the 'whole-minus-the-sum' (Mediano et al., 2019b). The expression $I\left(X_{t;}\, X_{t+1}\right)$ is the time-delayed mutual information (TDMI) (Barrett and Seth, 2011) and it quantifies the information flow from the past state to the future state. However, this measure is limited in that it only reflects interactions between pairs of variables,

that is between a target and a source. To expand on the capabilities for Φ for multi-source interactions, a framework known as Partial Information Decomposition (PID) was developed by (Williams and Beer, 2010). PID decomposes the total information that two sources give about a target into four distinct '*partial information atoms'* namely *synergistic, redundant*, and 2 *unique* atoms. This framework was further expanded to account for multivariate interactions with ΦID where each ΦID atom is denoted as a pair of PID atoms (Rosas et al., 2019), and subsequently a modified measure Φ^R was proposed to capture just the synergy and transfer (unique information flow from one component to another) components of integrated information (Mediano et al., 2021, 2019a).

The definition of Φ for a system is the *effective information* φ beyond its *Minimum Information Bipartition* (MIB) (Barrett and Seth, 2011). Effective information is a measure of how much better a system X is at predicting its own future after a time τ when it is considered as whole compared to when it is considered as the sum of two subsystems M^1 and M^2. Simply put, φ indicates how much predictive information is generated by the system over and above the predictive information generated by the two subsystems.

$$\varphi[X;\tau,\mathcal{B}] = I(X_{t-\tau};X_t) - \sum_{k=1}^{2} I(M_{t-\tau}^k\,;M_t^k) \tag{S.15}$$

where $\mathcal{B} = \{M^1, M^2\}$ is a given bipartition, I is Shannon's mutual information, and τ is the *integration timescale*.

A system's MIB is the bipartition with lowest φ, that is, the bipartition with maximal independence and therefore minimal mutual information. And so, we get

$$\Phi[X;\tau] = \varphi[X;\tau,\mathcal{B}^{MIB}] \tag{S.16}$$

$$\mathcal{B}^{MIB} = arg_{\mathcal{B}} min \frac{\varphi[X;\tau,\mathcal{B}]}{K(\mathcal{B})} \tag{S.17}$$

$$K(\mathcal{B}) = min\{H(M^1), H(M^2)\} \tag{S.18}$$

Where K is a normalization factor introduced to avoid biasing Φ to excessively unbalanced bipartitions (Mediano et al., 2022, 2016).

Returning to Φ^R , the integrated information without the redundancy components can then be expressed as:

$$\Phi^R[X;\tau] = \varphi[X;\tau,\mathcal{B}^{MIB}] + \min_{i,j} I(M^i_{t-\tau}; M^j_t) \tag{S.19}$$

where the Minimum Mutual Information (MMI) (Barrett, 2015) redundancy function is added back to the formulation.

Coalition configuration

As with any information theoretic measure, Φ^R is substrate-agnostic, that is, the relevant quantity for the calculation of Φ^R is some informational state of the system, and not the physical state of the system. We therefore define an informational state mapping upon which to calculate Φ^R. To this end, we use the coalition configuration of the system as the informational state reflecting the set of communities that are highly internally synchronized (Mediano et al., 2016).

$$X^c_t = f(x) = \begin{cases} 1, & if\ \ SYNC_{\psi}(t) > \gamma) \\ 0, & otherwise \end{cases} \tag{S.20}$$

where γ is the *coalition threshold*. The history of the system is now reduced to 5 interdependent binary time series. In this study we set γ = 0.8.

Coalition Entropy

Although $\langle \mathrm{META} \rangle_{\psi}$ and $\langle CHI \rangle_{G_T}$ detect the occurrence of cluster synchronization, neither indicate if the system visits a small number of chimera configurations repeatedly, or if the system has a large repertoire of such modes. To quantify the diversity of cluster synchronization, we calculate the coalition entropy (Mediano et al., 2016; Shanahan, 2010; Wildie and Shanahan, 2012).

$$H_c = -\sum_{i=1}^{S} p(s_i) log_2 p(s_i) \tag{S.21}$$

where S is the set of distinct coalitions the system can generate and $p(s)$ is the probability of coalition s arising at any timepoint t. With 5 communities $S = 2^5 = 32$.

References

Alonso Martínez, S., Deco, G., Ter Horst, G.J., Cabral, J., 2020. The Dynamics of Functional Brain Networks Associated With Depressive Symptoms in a Nonclinical Sample. Front. Neural Circuits 14, 570583. https://doi.org/10.3389/fncir.2020.570583

Arbabyazd, L.M., Lombardo, D., Blin, O., Didic, M., Battaglia, D., Jirsa, V., 2020. Dynamic Functional Connectivity as a complex random walk: Definitions and the dFCwalk toolbox. MethodsX 7, 101168. https://doi.org/10.1016/j.mex.2020.101168

Arenas, A., Díaz-Guilera, A., Kurths, J., Moreno, Y., Zhou, C., 2008. Synchronization in complex networks. Phys. Rep. 469, 93–153. https://doi.org/10.1016/j.physrep.2008.09.002

Balduzzi, D., Tononi, G., 2008. Integrated Information in Discrete Dynamical Systems: Motivation and Theoretical Framework. PLOS Comput. Biol. 4, e1000091. https://doi.org/10.1371/journal.pcbi.1000091

Barrett, A.B., 2015. Exploration of synergistic and redundant information sharing in static and dynamical Gaussian systems. Phys. Rev. E 91, 052802. https://doi.org/10.1103/PhysRevE.91.052802

Barrett, A.B., Seth, A.K., 2011. Practical Measures of Integrated Information for Time-Series Data. PLOS Comput. Biol. 7, e1001052. https://doi.org/10.1371/journal.pcbi.1001052

Battaglia, D., Boudou, T., Hansen, E.C.A., Lombardo, D., Chettouf, S., Daffertshofer, A., McIntosh, A.R., Zimmermann, J., Ritter, P., Jirsa, V., 2020. Dynamic Functional Connectivity between order and randomness and its evolution across the human adult lifespan. NeuroImage 222, 117156. https://doi.org/10.1016/j.neuroimage.2020.117156

Botcharova, M., 2014. Modelling and analysis of amplitude, phase and synchrony in human brain activity patterns.

Cabral, J., Hugues, E., Sporns, O., Deco, G., 2011. Role of local network oscillations in resting-state functional connectivity. NeuroImage 57, 130–139. https://doi.org/10.1016/j.neuroimage.2011.04.010

Cabral, J., Luckhoo, H., Woolrich, M., Joensson, M., Mohseni, H., Baker, A., Kringelbach, M.L., Deco, G., 2014. Exploring mechanisms of spontaneous functional connectivity in MEG: How delayed network interactions lead to

structured amplitude envelopes of band-pass filtered oscillations. NeuroImage 90, 423–435. https://doi.org/10.1016/j.neuroimage.2013.11.047

Cabral, J., Vidaurre, D., Marques, P., Magalhães, R., Silva Moreira, P., Miguel Soares, J., Deco, G., Sousa, N., Kringelbach, M.L., 2017. Cognitive performance in healthy older adults relates to spontaneous switching between states of functional connectivity during rest. Sci. Rep. 7, 1–13. https://doi.org/10.1038/s41598-017-05425-7

Cai, B., Zhou, Z., Zhang, A., Zhang, G., Xiao, L., Stephen, J.M., Wilson, T.W., Calhoun, V.D., Wang, Y.-P., 2022. Functional connectomes incorporating phase synchronization for the characterization and prediction of individual differences. J. Neurosci. Methods 372, 109539. https://doi.org/10.1016/j.jneumeth.2022.109539

Chang, C., Glover, G.H., 2010. Time–frequency dynamics of resting-state brain connectivity measured with fMRI. NeuroImage 50, 81–98. https://doi.org/10.1016/j.neuroimage.2009.12.011

Cheng, J.C., Anzolin, A., Berry, M., Honari, H., Paschali, M., Lazaridou, A., Lee, J., Ellingsen, D.-M., Loggia, M.L., Grahl, A., Lindquist, M.A., Edwards, R.R., Napadow, V., 2021. Dynamic Functional Brain Connectivity Underlying Temporal Summation of Pain in Fibromyalgia. Arthritis Rheumatol. 74, 700–710. https://doi.org/10.1002/art.42013

Daffertshofer, A., Ton, R., Kringelbach, M.L., Woolrich, M., Deco, G., 2018. Distinct criticality of phase and amplitude dynamics in the resting brain. NeuroImage, Brain Connectivity Dynamics 180, 442–447. https://doi.org/10.1016/j.neuroimage.2018.03.002

Deco, G., Cabral, J., Woolrich, M.W., Stevner, A.B.A., van Hartevelt, T.J., Kringelbach, M.L., 2017. Single or multiple frequency generators in on-going brain activity: A mechanistic whole-brain model of empirical MEG data. NeuroImage 152, 538–550. https://doi.org/10.1016/j.neuroimage.2017.03.023

Deco, G., Kringelbach, M.L., 2016. Metastability and Coherence: Extending the Communication through Coherence Hypothesis Using A Whole-Brain Computational Perspective. Trends Neurosci. 39, 125–135. https://doi.org/10.1016/j.tins.2016.01.001

Eke, A., Hermán, P., Bassingthwaighte, J., Raymond, G., Percival, D., Cannon, M., Balla, I., Ikrényi, C., 2000. Physiological time series: distinguishing fractal noises from motions. Pflüg. Arch. 439, 403–415. https://doi.org/10.1007/s004249900135

Eke, A., Herman, P., Kocsis, L., Kozak, L.R., 2002. Fractal characterization of complexity in temporal physiological signals. Physiol. Meas. 23, R1–R38. https://doi.org/10.1088/0967-3334/23/1/201

Figueroa, C.A., Cabral, J., Mocking, R.J.T., Rapuano, K.M., Hartevelt, T.J. van, Deco, G., Expert, P., Schene, A.H., Kringelbach, M.L., Ruhé, H.G., 2019. Altered ability to access a clinically relevant control network in patients remitted from major depressive disorder. Hum. Brain Mapp. 40, 2771–2786. https://doi.org/10.1002/hbm.24559

Glerean, E., Salmi, J., Lahnakoski, J.M., Jääskeläinen, I.P., Sams, M., 2012. Functional Magnetic Resonance Imaging Phase Synchronization as a Measure of Dynamic Functional Connectivity. Brain Connect. 2, 91–101. https://doi.org/10.1089/brain.2011.0068

Haken, H., 1989. Synergetics: an overview. Rep. Prog. Phys. 52, 515–553. https://doi.org/10.1088/0034-4885/52/5/001

Hansen, E.C.A., Battaglia, D., Spiegler, A., Deco, G., Jirsa, V.K., 2015. Functional connectivity dynamics: Modeling the switching behavior of the resting state. NeuroImage 105, 525–535. https://doi.org/10.1016/j.neuroimage.2014.11.001

Hardstone, R., Poil, S.-S., Schiavone, G., Jansen, R., Nikulin, V.V., Mansvelder, H.D., Linkenkaer-Hansen, K., 2012. Detrended Fluctuation Analysis: A Scale-Free View on Neuronal Oscillations. Front. Physiol. 0. https://doi.org/10.3389/fphys.2012.00450

Hellyer, P.J., Shanahan, M., Scott, G., Wise, R.J.S., Sharp, D.J., Leech, R., 2014. The Control of Global Brain Dynamics: Opposing Actions of Frontoparietal Control and Default Mode Networks on Attention. J. Neurosci. 34, 451–461. https://doi.org/10.1523/JNEUROSCI.1853-13.2014

Honari, H., Choe, A.S., Lindquist, M.A., 2021. Evaluating phase synchronization methods in fMRI: A comparison study and new approaches. NeuroImage 228, 117704. https://doi.org/10.1016/j.neuroimage.2020.117704

Kakihara, Y., 2003. Stochastic Processes, in: Meyers, R.A. (Ed.), Encyclopedia of Physical Science and Technology (Third Edition). Academic Press, New York, pp. 105–116. https://doi.org/10.1016/B0-12-227410-5/00739-0

Lee, W.H., Doucet, G.E., Leibu, E., Frangou, S., 2018. Resting-state network connectivity and metastability predict clinical symptoms in schizophrenia. Schizophr. Res. 201, 208–216. https://doi.org/10.1016/j.schres.2018.04.029

Lee, W.H., Moser, D.A., Ing, A., Doucet, G.E., Frangou, S., 2019. Behavioral and Health Correlates of Resting-State Metastability in the Human Connectome Project. Brain Topogr. 32, 80–86. https://doi.org/10.1007/s10548-018-0672-5

Lombardo, D., Cassé-Perrot, C., Ranjeva, J.-P., Le Troter, A., Guye, M., Wirsich, J., Payoux, P., Bartrés-Faz, D., Bordet, R., Richardson, J.C., Felician, O., Jirsa, V., Blin, O., Didic, M., Battaglia, D., 2020. Modular slowing of resting-state dynamic functional connectivity as a marker of cognitive dysfunction induced by sleep deprivation. NeuroImage 222, 117155. https://doi.org/10.1016/j.neuroimage.2020.117155

Lord, L.-D., Expert, P., Atasoy, S., Roseman, L., Rapuano, K., Lambiotte, R., Nutt, D.J., Deco, G., Carhart-Harris, R.L., Kringelbach, M.L., Cabral, J., 2019. Dynamical exploration of the repertoire of brain networks at rest is modulated by psilocybin. NeuroImage 199, 127–142. https://doi.org/10.1016/j.neuroimage.2019.05.060

Luppi, A.I., Mediano, P.A.M., Rosas, F.E., Allanson, J., Pickard, J.D., Carhart-Harris, R.L., Williams, G.B., Craig, M.M., Finoia, P., Owen, A.M., Naci, L., Menon, D.K., Bor, D., Stamatakis, E.A., 2020. A Synergistic Workspace for Human Consciousness Revealed by Integrated Information Decomposition. bioRxiv 2020.11.25.398081. https://doi.org/10.1101/2020.11.25.398081

Maaten, L.V.D., Hinton, G.E., 2008. Visualizing Data using t-SNE. J. Mach. Learn. 2579–2605.

Mediano, P.A.M., Farah, J.C., Shanahan, M., 2016. Integrated Information and Metastability in Systems of Coupled Oscillators. ArXiv160608313 Q-Bio.

Mediano, P.A.M., Rosas, F., Carhart-Harris, R.L., Seth, A.K., Barrett, A.B., 2019a. Beyond integrated information: A taxonomy of information dynamics phenomena. ArXiv190902297 Phys. Q-Bio.

Mediano, P.A.M., Rosas, F.E., Farah, J.C., Shanahan, M., Bor, D., Barrett, A.B., 2022. Integrated information as a common signature of dynamical and information-processing complexity. Chaos Interdiscip. J. Nonlinear Sci. 32, 013115. https://doi.org/10.1063/5.0063384

Mediano, P.A.M., Rosas, F.E., Luppi, A.I., Carhart-Harris, R.L., Bor, D., Seth, A.K., Barrett, A.B., 2021. Towards an extended taxonomy of information dynamics via Integrated Information Decomposition. ArXiv210913186 Phys. Q-Bio.

Mediano, P.A.M., Seth, A.K., Barrett, A.B., 2019b. Measuring Integrated Information: Comparison of Candidate Measures in Theory and Simulation. Entropy 21, 17. https://doi.org/10.3390/e21010017

Metzler, R., Jeon, J.-H., Cherstvy, A.G., Barkai, E., 2014. Anomalous diffusion models and their properties: non-stationarity, non-ergodicity, and ageing at the centenary of single particle tracking. Phys Chem Chem Phys 16, 24128–24164. https://doi.org/10.1039/C4CP03465A

Niazy, R.K., Xie, J., Miller, K., Beckmann, C.F., Smith, S.M., 2011. Chapter 17 - Spectral characteristics of resting state networks, in: Van Someren, E.J.W., Van Der Werf, Y.D., Roelfsema, P.R., Mansvelder, H.D., Lopes Da Silva, F.H. (Eds.), Progress in Brain Research, Slow Brain Oscillations of Sleep, Resting State and Vigilance. Elsevier, pp. 259–276. https://doi.org/10.1016/B978-0-444-53839-0.00017-X

Nobukawa, S., Kikuchi, M., Takahashi, T., 2019. Changes in functional connectivity dynamics with aging: A dynamical phase synchronization approach. NeuroImage 188, 357–368. https://doi.org/10.1016/j.neuroimage.2018.12.008

Pederson, 2018. On the relationship between instantaneous phase synchrony and correlation-based sliding windows for time-resolved fMRI connectivity analysis | Elsevier Enhanced Reader [WWW Document]. https://doi.org/10.1016/j.neuroimage.2018.06.020

Ponce-Alvarez, A., Deco, G., Hagmann, P., Romani, G.L., Mantini, D., Corbetta, M., 2015. Resting-State Temporal Synchronization Networks Emerge from Connectivity Topology and Heterogeneity. PLOS Comput. Biol. 11, e1004100. https://doi.org/10.1371/journal.pcbi.1004100

Rosas, F.E., Mediano, P.A.M., Gastpar, M., Jensen, H.J., 2019. Quantifying high-order interdependencies via multivariate extensions of the mutual information. Phys. Rev. E 100, 032305. https://doi.org/10.1103/PhysRevE.100.032305

Sendi, M.S.E., Zendehrouh, E., Fu, Z., Liu, J., Du, Y., Mormino, E., Salat, D.H., Calhoun, V.D., Miller, R.L., 2021. Disrupted dynamic functional network connectivity among cognitive control networks in the progression of Alzheimer’s disease. bioRxiv 2020.12.31.424877. https://doi.org/10.1101/2020.12.31.424877

Shanahan, M., 2010. Metastable chimera states in community-structured oscillator networks. Chaos Interdiscip. J. Nonlinear Sci. 20, 013108. https://doi.org/10.1063/1.3305451

Tagliazucchi, E., Chialvo, D.R., Siniatchkin, M., Amico, E., Brichant, J.-F., Bonhomme, V., Noirhomme, Q., Laufs, H., Laureys, S., 2016. Large-scale signatures of unconsciousness are consistent with a departure from critical

dynamics. J. R. Soc. Interface 13, 20151027. https://doi.org/10.1098/rsif.2015.1027

Tagliazucchi, E., von Wegner, F., Morzelewski, A., Brodbeck, V., Jahnke, K., Laufs, H., 2013. Breakdown of long-range temporal dependence in default mode and attention networks during deep sleep. Proc. Natl. Acad. Sci. 110, 15419–15424. https://doi.org/10.1073/pnas.1312848110

Tetereva, A., Kartashov, S., Ivanitsky, A., Martynova, O., 2020. Variance and Scale-Free Properties of Resting-State Blood Oxygenation Level-Dependent Signal After Fear Memory Acquisition and Extinction. Front. Hum. Neurosci. 14, 509075. https://doi.org/10.3389/fnhum.2020.509075

Tognoli, E., Kelso, J.A.S., 2014. The Metastable Brain. Neuron 81, 35–48. https://doi.org/10.1016/j.neuron.2013.12.022

Ton, R., Daffertshofer, A., 2016. Model selection for identifying power-law scaling. NeuroImage 136, 215–226. https://doi.org/10.1016/j.neuroimage.2016.01.008

Tononi, G., Sporns, O., Edelman, G.M., 1994. A measure for brain complexity: relating functional segregation and integration in the nervous system. Proc. Natl. Acad. Sci. 91, 5033–5037. https://doi.org/10.1073/pnas.91.11.5033

Vohryzek, J., Deco, G., Cessac, B., Kringelbach, M.L., Cabral, J., 2020. Ghost Attractors in Spontaneous Brain Activity: Recurrent Excursions Into Functionally-Relevant BOLD Phase-Locking States. Front. Syst. Neurosci. 14, 20. https://doi.org/10.3389/fnsys.2020.00020

Wang, X.-H., Li, L., Xu, T., Ding, Z., 2015. Investigating the Temporal Patterns within and between Intrinsic Connectivity Networks under Eyes-Open and Eyes-Closed Resting States: A Dynamical Functional Connectivity Study Based on Phase Synchronization. PLOS ONE 10, e0140300. https://doi.org/10.1371/journal.pone.0140300

Wildie, M., Shanahan, M., 2012. Metastability and chimera states in modular delay and pulse-coupled oscillator networks. Chaos Interdiscip. J. Nonlinear Sci. 22, 043131. https://doi.org/10.1063/1.4766592

Williams, P.L., Beer, R.D., 2010. Nonnegative Decomposition of Multivariate Information. ArXiv10042515 Math-Ph Physicsphysics Q-Bio.

Yang, H., Shew, W.L., Roy, R., Plenz, D., 2012. Maximal Variability of Phase Synchrony in Cortical Networks with Neuronal Avalanches. J. Neurosci. 32, 1061–1072. https://doi.org/10.1523/JNEUROSCI.2771-11.2012
